# *M*In(HAsO_4_)_2_ (*M* = K, Rb, Cs): three new hydrogen­arsenates adopting two different structure types

**DOI:** 10.1107/S205698901701355X

**Published:** 2017-09-29

**Authors:** Karolina Schwendtner, Uwe Kolitsch

**Affiliations:** a Institute for Chemical Technology and Analytics, Division of Structural Chemistry, TU Wien, Getreidemarkt 9/164-SC, 1060 Vienna, Austria; b Mineralogisch-Petrographische Abteilung, Naturhistorisches Museum Wien, Burgring 7, 1010 Wien, and Institut für Mineralogie und Kristallographie, Universität Wien, Althanstrasse 14, 1090 Wien, Austria

**Keywords:** crystal structure, KIn(HAsO_4_)_2_, RbIn(HAsO_4_)_2_, CsIn(HAsO_4_)_2_, arsenate

## Abstract

Potassium indium bis­[hydrogen arsenate(V)], KIn(HAsO_4_)_2_, rubidium indium bis­[hydrogen arsenate(V)], RbIn(HAsO_4_)_2_, and caesium indium bis­[hydrogen arsenate(V)], CsIn(HAsO_4_)_2_, were grown under mild hydro­thermal conditions (*T* = 493 K, 7–8 d). KIn(HAsO_4_)_2_ adopts the KSc(HAsO_4_)_2_ structure type (space group *C*2/*c*), while RbIn(HAsO_4_)_2_ and CsIn(HAsO_4_)_2_ crystallize in the space group *R*-3*c* and are the first arsenate representatives of the RbFe(HPO_4_)_2_ structure type. All three compounds have tetra­hedral–octa­hedral framework topologies. The *M* ^+^ cations, located in voids of the respective framework, are slightly disordered in RbIn(HAsO_4_)_2_. In KIn(HAsO_4_)_2_, there is a second K-atom position with a very low occupancy, which may suggest that the K atom can easily move in the channels extending along [101].

## Abstract 2

KIn(HAsO_4_)_2_ adopts the KSc(HAsO_4_)_2_ structure type (space group *C*2/*c*), while RbIn(HAsO_4_)_2_ and CsIn(HAsO_4_)_2_ crystallize in the space group *R*-3*c* and are the first arsenate representatives of the RbFe(HPO_4_)_2_ structure type. All three compounds have tetra­hedral–octa­hedral framework topologies. The *M* ^+^ cations, located in voids of the respective framework, are slightly disordered in RbIn(HAsO_4_)_2_. In KIn(HAsO_4_)_2_, there is a second K-atom position with a very low occupancy, which may suggest that the K atom can easily move in the channels extending along [101].

## Chemical context   

Metal arsenates often form tetra­hedral–octa­hedral framework structures that frequently show potentially inter­esting properties, such as ion conductivity, ion exchange and catalytic properties (Masquelier *et al.*, 1990 ▸, 1994*a* ▸,*b* ▸, 1995 ▸, 1996 ▸, 1998 ▸; Mesa *et al.*, 2000 ▸; Ouerfelli *et al.*, 2007*a* ▸,*b* ▸, 2008 ▸; Pintard-Scrépel *et al.*, 1983 ▸; Rousse *et al.*, 2013 ▸). In the course of a detailed study of the system *M* ^+^–*M* ^3+^–As–O–(H) by hydro­thermal syntheses, a large variety of new arsenate(V) compounds and structure types were found (Kolitsch, 2004 ▸; Schwendtner, 2006 ▸; Schwendtner & Kolitsch, 2004*a* ▸,*b* ▸, 2005 ▸, 2007*a* ▸,*b* ▸,*c* ▸,*d* ▸, 2017*a* ▸,*b* ▸,*c* ▸).

The three new title compounds belong to the family of hydrogenarsenate compounds with the general formula *M* ^+^ *M* ^3+^(HAsO_4_)_2_. Including the three compounds reported here, nine compounds with this general formula are known. They crystallize in four different structure types. KIn(HAsO_4_)_2_ is a further representative of the KSc(HAsO_4_)_2_ structure type (Schwendtner & Kolitsch, 2004*a* ▸), which is also adopted by AgGa(HAsO_4_)_2_ and AgAl(HAsO_4_)_2_ (Schwendtner & Kolitsch, 2017*c* ▸). The (H_3_O)Fe(HPO_4_)_2_ structure type (Vencato *et al.*, 1989 ▸) is adopted by CsSc(HAsO_4_)_2_ (Schwendtner & Kolitsch, 2004 ▸b). Another modification of CsSc(HAsO_4_)_2_ crystallizes in the (NH_4_)Fe(HPO_4_)_2_ type (Yakubovich, 1993 ▸), in which also (NH_4_)Fe(HAsO_4_)_2_ crystallizes (Ouerfelli *et al.*, 2014 ▸). The two new title compounds RbIn(HAsO_4_)_2_ and CsIn(HAsO_4_)_2_ adopt a structure type hitherto unknown among arsenates which is, however, known from the phosphates RbFe(HPO_4_)_2_ (Lii & Wu, 1994 ▸) and Rb*M* ^3+^(HPO_4_)_2_ (*M* = Al, Ga) (Lesage *et al.*, 2007 ▸). All of these compounds consist of frameworks of singly protonated AsO_4_ tetra­hedra and *M* ^3+^O_6_ octa­hedra. The *M* ^+^ cations occupy channels that extend along one or more directions in the framework.

A number of *M* ^+^–In–arsenates have been reported in the literature. Among these are several diarsenates: NaInAs_2_O_7_ (Belam *et al.*, 1997 ▸), KInAs_2_O_7_ (Schwendtner & Kolitsch, 2017*b* ▸) and RbInAs_2_O_7_, TlInAs_2_O_7_ and (NH_4_)InAs_2_O_7_ (Schwendtner, 2006 ▸), furthermore Na_3_In_2_(AsO_4_)_3_ (Lii & Ye, 1997 ▸; Khorari *et al.*, 1997 ▸) and KIn(H_2_O)(H_1.5_AsO_4_)_2_(H_2_AsO_4_) (Schwendtner & Kolitsch, 2007*c* ▸). There also exist indexed X-ray powder diffraction data of Li_3_In_2_(AsO_4_)_3_ (Winand *et al.*, 1990 ▸) and unindexed powder patterns of KIn(HAsO_4_)_2_·*x*H_2_O, RbIn(HAsO_4_)_2_·*x*H_2_O, CsIn(HAsO_4_)_2_·*x*H_2_O and CsInAs_2_O_7_ (Ezhova *et al.*, 1977 ▸).

The hydrogenphosphates KIn(HPO_4_)_2_ and RbIn(HPO_4_)_2_ (Filaretov *et al.*, 2002*b* ▸), which are the phosphate analogues of two of the title compounds, crystallize in the (NH_4_)In(HPO_4_)_2_ structure type (*P*2_1_/*c*; Filaretov *et al.*, 2002*a* ▸; Mao *et al.*, 2002 ▸), for which no arsenate members were known prior to the present work. CsIn(HPO_4_)_2_ (Huang *et al.*, 2004 ▸; Lesage *et al.*, 2007 ▸) is known as two modifications, the (NH_4_)Fe(HPO_4_)_2_-type (*P* 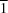; Yakubovich, 1993 ▸) and the (H_3_O)Fe(HPO_4_)_2_-type (*P*2_1_/*c*; Vencato *et al.*, 1989 ▸). Both structure types are common among hydrogenphosphates, with eleven and seven members, respectively, and both have one arsenate representative each, *viz*. α- and β-CsSc(HAsO_4_)_2_ (Schwendtner & Kolitsch, 2004 ▸). The (NH_4_)Fe(HPO_4_)_2_-type CsIn(HPO_4_)_2_ is closely related to and basically a distorted variety of the RbFe(HPO_4_)_2_ type in which CsIn(HAsO_4_)_2_ crystallizes (see discussion in Lesage *et al.*, 2007 ▸). According to Huang *et al.* (2004 ▸), a second variety of RbIn(HPO_4_)_2_ exists, which is also isotypic to (H_3_O)Fe(HPO_4_)_2_.

## Structural commentary   

**Figure 1:**
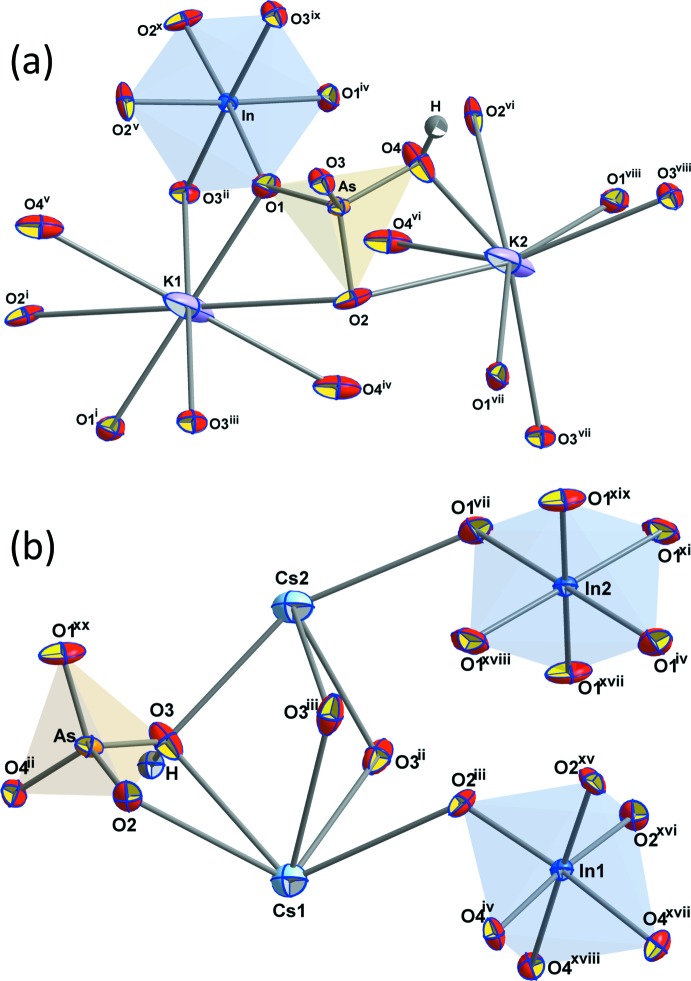
The principal building units of (*a*) KIn(HAsO_4_)_2_ and (*b*) CsIn(HAsO_4_)_2_, shown as displacement ellipsoids at the 70% probability level. Symmetry codes: KIn(HAsO_4_)_2_: (i) −*x* + 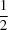, −*y* + 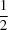, −*z*; (ii) −*x*, −*y* + 1, −*z*; (iii) *x* + 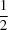, *y* − 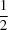, *z*; (iv) *x* − 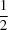, −*y* + 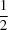, *z* − 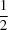; (v) −*x* + 1, *y*, −*z* + 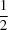; (vi) *x*, −*y* + 1, *z* − 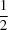; (vii) −*x* + 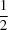, *y* − 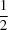, −*z* + 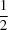; (viii) −*x*, *y*, −*z* + 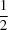; (ix) −*x* + 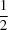, *y* + 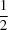, −*z* + 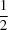; (*x*) *x* − 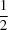, *y* + 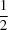, *z*; CsIn(HAsO_4_)_2_: (ii) −*x*, −*x* + *y*, −*z* + 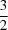; (iii) −*x* + *y*, −*x*, *z*; (iv) −*y*, *x* − *y*, *z*; (vii) *y* + 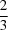, −*x* + *y* + 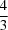, −*z* + 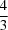; (xi) *x* − *y* − 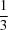, *x* + 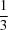, −*z* + 4/3; (xv) −*y*, *x* − *y* + 1, *z*; (xvi) *x* + 1, *y* + 1, *z*; (xvii) *x*, *y* + 1, *z*; (xviii) −*x* + *y*+1, −*x* + 1, *z*; (xix) −*x* + 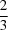, −*y* + 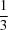, −*z* + 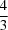; (xx) *x* − 1, *y*, *z*.

**Figure 2:**
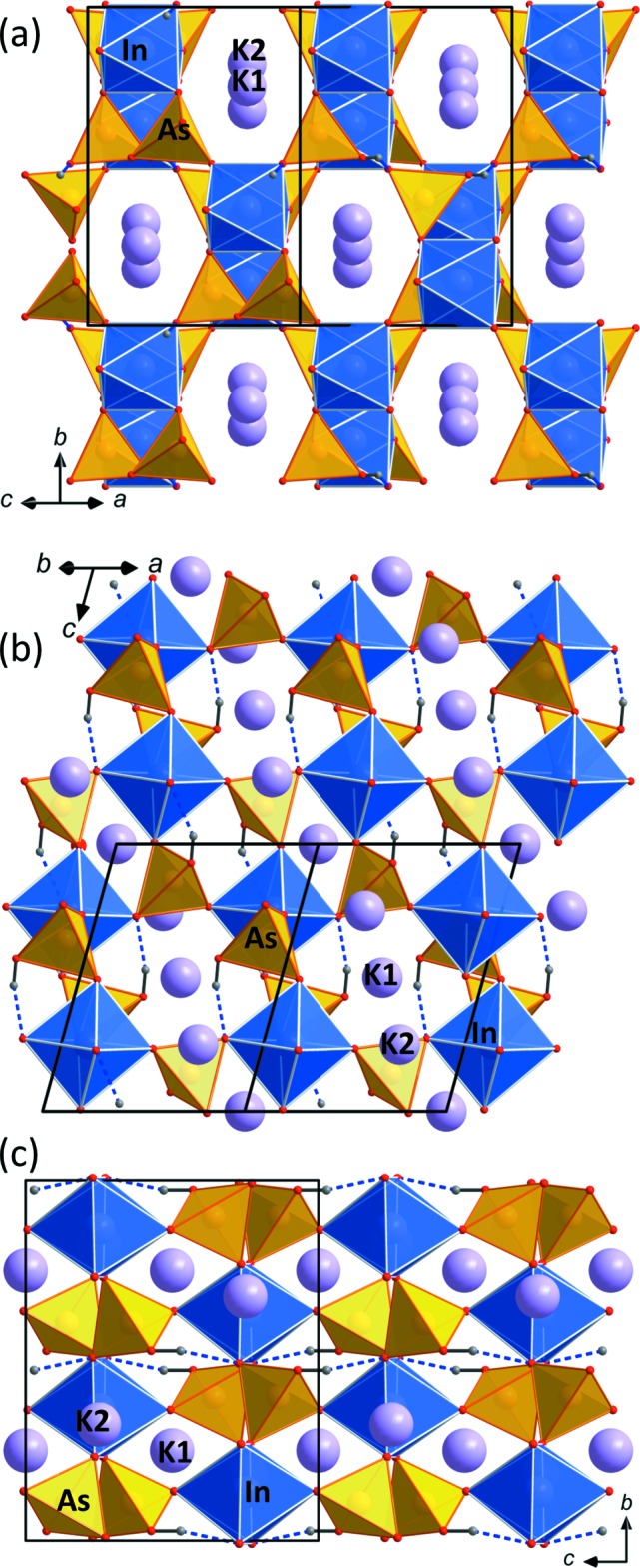
The framework structure of KIn(HAsO_4_)_2_ in views parallel to (*a*) [101], (*b*) [110] and (*c*) [100]. The K atoms are located in channels of the framework (note that the K2 position has an occupancy of only 0.024 (2). Hydrogen bonds (dashed lines) reinforce the framework and extend roughly along *c*.

KIn(HAsO_4_)_2_ crystallizes in space group *C*2/*c* and is isotypic to KSc(HAsO_4_)_2_ (Schwendtner & Kolitsch, 2004 ▸ *a*), AgGa(HAsO_4_)_2_ and AgAl(HAsO_4_)_2_ (Schwendtner & Kolitsch, 2017*c* ▸). The asymmetric unit contains one K, one In, one As, one H and four O atoms (Fig. 1 ▸ *a*). The slightly distorted InO_6_ octa­hedra share corners with six HAsO_4_ tetra­hedra, thus forming a three-dimensional anionic framework with narrow channels parallel to [110] and [101] (Fig. 2 ▸ *a*,*b*) which host the K atoms. There are two K-atom positions (K1 and K2), at a distance of 2.653 (15) Å from each other. The K1 position is located on an inversion centre and has a refined occupancy of 0.976 (2), while K2, which lies between two K1 positions, is located on a twofold axis (like the In atom) and has a refined occupancy of 0.024 (2). Both K-atom positions show a [4 + 4]-coordination with average K—O bond lengths of 2.949 and 3.016 Å for K1 and K2, respectively (Table 1 ▸). This is slightly longer than the reported average K—O bond length for ^[8]^K atoms of 2.85 Å (Baur, 1981 ▸). However, bond-valence calculations after Gagné & Hawthorne (2015 ▸) show bond-valence sums (BVSs) of 0.99 valence units (v.u.) for K1 and 0.85 v.u. for K2, indicating an ‘underbonded’ character of K2, and explaining the difference in site occupancies.

As expected, the protonated AsO_4_ tetra­hedron is strongly distorted as three vertices connect to neighbouring InO_6_ octa­hedra, while O4 (OH) is a terminal vertex and only involved in a medium–strong hydrogen bond (Fig. 2 ▸ *b* and 2*c*; Table 4 ▸).

Calculated BVSs (Gagné & Hawthorne, 2015 ▸) of the framework atoms amount to 3.06 v.u. for In, 5.07 v.u. for As and 2.11/1.83/1.96/1.20 v.u. for O1–O4, respectively. Although these sums appear slightly too high for In and As, the average In—O and As—O bond lengths fit very well to published averages: the average As—O bond length in KIn(HAsO_4_)_2_ is 1.682 Å and the As—OH bond length is 1.723 Å, very close to the average of 704 analyzed AsO_4_ groups in inorganic compounds [1.686 (10) Å; Schwendtner, 2008 ▸] and the average As—OH in 45 HAsO_4_ groups [1.72 (3) Å; Schwendtner, 2008 ▸], respectively. The average In—O bond length (2.132 Å) is slightly shorter than the published average of 2.141 Å for inorganic compounds (Baur, 1981 ▸).

**Figure 3:**
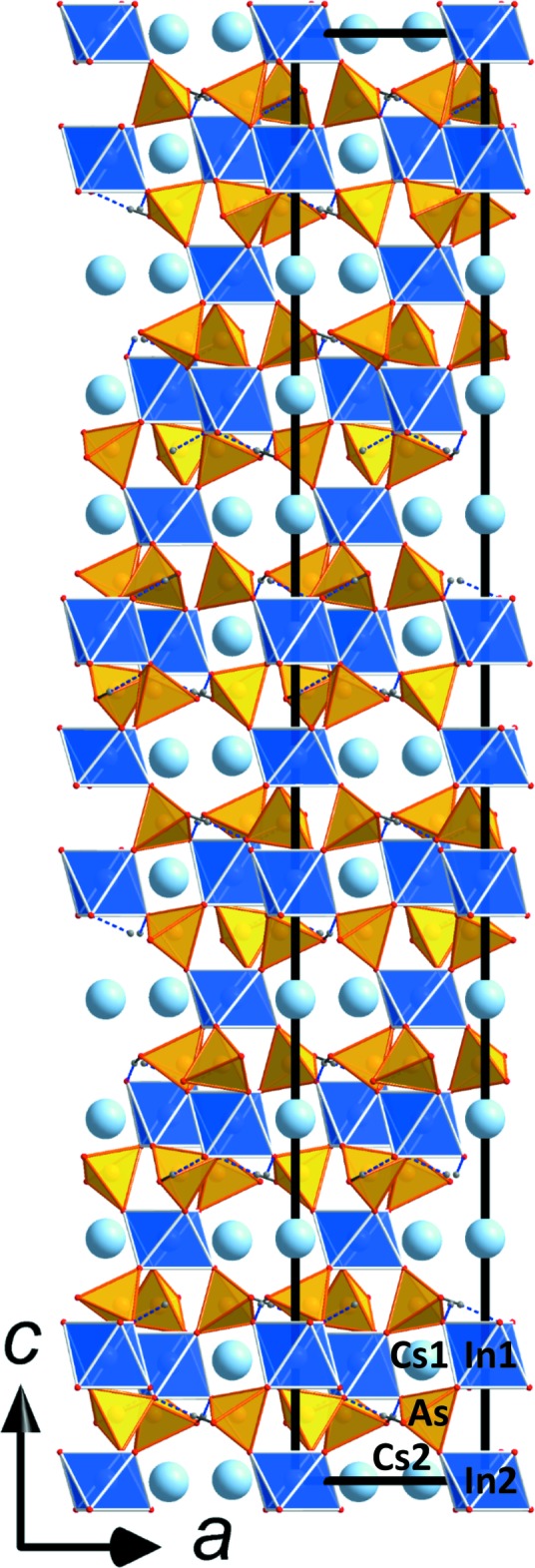
The framework structure of CsIn(HAsO_4_)_2_ in a view parallel to *b*. The unit cell (outlined) is characterized by a long *c* axis. Cs atoms occupy channels extending parallel to *a* and *b*. Hydrogen bonds are shown as dashed lines.

**Figure 4:**
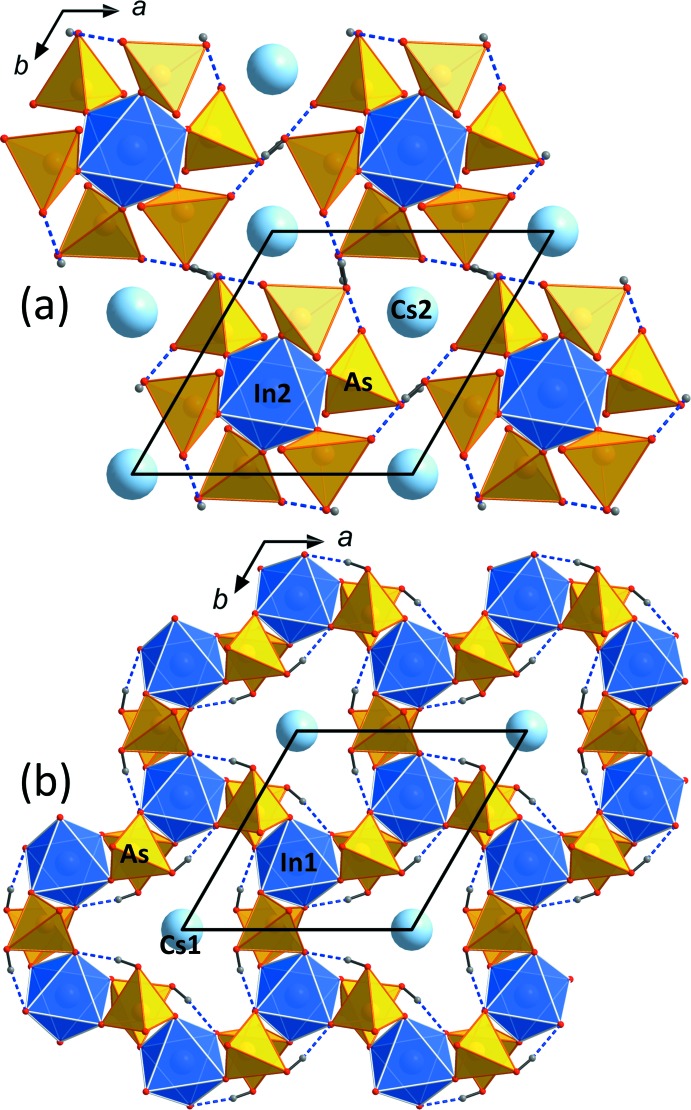
View along *c* of the two different layers involving the two different Cs atoms positions in the framework structure of CsIn(HAsO_4_)_2_. These layers are stacked along *c* (*cf*. Fig. 3 ▸). Hydrogen bonds are shown as dashed lines.

RbIn(HAsO_4_)_2_ and CsIn(HAsO_4_)_2_ crystallize in the space group *R* 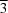 *c* and are isotypic to RbFe(HPO_4_)_2_ (Lii & Wu, 1994 ▸) and Rb*M* ^3+^(HPO_4_)_2_ (*M* = Al, Ga) (Lesage *et al.*, 2007 ▸). The asymmetric unit contains two *M* ^+^, two In, one As, one H and four O positions and the structure is characterized by a long *c* axis in the hexa­gonal setting (Fig. 3 ▸). As in KIn(HAsO_4_)_2_, each InO_6_ octa­hedron shares six vertices with six HAsO_4_ tetra­hedra, resulting in an InAs_6_O_24_ group. These groups are in turn connected *via* three corners to other InO_6_ octa­hedra. The protonated apices of the HAsO_4_ tetra­hedra form a strong hydrogen bond (O—H⋯O = 2.62–2.63 Å) to the neighbouring InAs_6_O_24_ group. The InAs_6_O_24_ groups in RbIn(HAsO_4_)_2_ and CsIn(HAsO_4_)_2_ are arranged in layers normal to *c*, and the groups within these layers are inter­connected by strong hydrogen bonds extending in directions [100] and [110] (Fig. 4 ▸ *a* and 4*b*). The 12-coordinated Cs atoms are located in channels which extend along *a* and *b*. As in KIn(HAsO_4_)_2_, the average In—O bond lengths (2.138/2.131 and 2.139/2.133 Å for In1/In2 in the Rb and Cs compounds, respectively; Tables 2 ▸ and 3 ▸) are slightly smaller than the literature value (2.141 Å; Baur, 1981 ▸), while the average As—O bond lengths (1.683 and 1.687 Å) show good agreement with the literature value (see above). The calculated BVSs (Gagné & Hawthorne, 2015 ▸) amount to 1.05 (Rb1), 0.65 (Rb2), 3.02 (In1), 3.07 (In2), 5.07 (As) and 1.94/1.90/1.30/1.82 v.u. (O1–O4) for RbIn(HAsO_4_)_2_, and 0.92 (Cs1), 0.80 (Cs2), 3.02 (In1), 3.05 (In2), 5.01 (As) and 1.94/1.88/1.29/1.80 v.u. (O1–O4) for CsIn(HAsO_4_)_2_. These values are reasonably close to ideal valencies, although the fairly low value for Rb2 is noteworthy; apparently the Rb2-hosting cavity is too large for the Rb atom. In fact, both Rb atoms seem to ‘rattle’ somewhat in their cavities and are characterized by rather large anisotropic displacement ellipsoids; therefore, they were modeled by split positions involving an additional, low-occupancy Rb position (Rb1*B*, Rb2*B*) in each case. The severely underbonded O3 atom is donor of the strong hydrogen bonds (Tables 5 ▸ and 6 ▸). As expected, the unit-cell volume of the isotypic phosphates is about 20% smaller than that of the arsenates. The stronger condensation due to the smaller stronger-bonded phosphate also leads to even stronger hydrogen bonds, with O—H⋯O distances ranging from 2.58 to 2.59 Å (Lii & Wu, 1994 ▸; Lesage *et al.*, 2007 ▸).

## Synthesis and crystallization   

The compounds were grown by hydro­thermal synthesis at 493 K (7–8 d, autogeneous pressure, slow furnace cooling) using Teflon-lined stainless steel autoclaves with an approximate filling volume of 2 cm^3^. Reagent-grade KOH/Rb_2_CO_3_/Cs_2_CO_3_, In_2_O_3_, α-Al_2_O_3_ (only in the case of the K–In–arsenate) and H_3_AsO_4_·0.5H_2_O were used as starting reagents in approximate volume ratios of *M* ^+^:*M* ^3+^:As of 1:1:2. In the synthesis of KIn(HAsO_4_)_2_, the In_2_O_3_:α-Al_2_O_3_ ratio was 1:1. The vessels were filled with distilled water to about 70% of their inner volumes which led to final pH values of < 1 for all synthesis batches except KIn(HAsO_4_)_2_ (initial pH 4.5, final pH 3). The reaction products were washed thoroughly with distilled water, filtered and dried at room temperature. They are stable in air.

**Figure 5:**
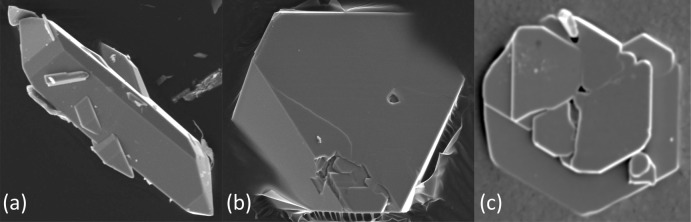
SEM micrographs of hydro­thermally synthesized crystals of (*a*) KIn(HAsO_4_)_2_, (*b*) RbIn(HAsO_4_)_2_ and (*c*) CsIn(HAsO_4_)_2_.

KIn(HAsO_4_)_2_ formed prismatic-bipyramidal crystals (Fig. 5 ▸ *a*) that were accompanied by cubic crystals of synthetic pharmacoalumite [KAl_4_(AsO_4_)_3_(OH)_4_·6.5H_2_O]. Thus, the Al and In present in the synthesis of these phases seemingly fractionate completely between the two phases KIn(HAsO_4_)_2_ and KAl_4_(AsO_4_)_3_(OH)_4_·6.5H_2_O. RbIn(HAsO_4_)_2_ and CsIn(HAsO_4_)_2_ formed pseudo-octa­hedral crystals and platelets with pseudohexa­gonal outline (Fig. 5 ▸ *b* and 5*c*, respectively). RbIn(HAsO_4_)_2_ was accompanied by crystals of RbInAs_2_O_7_ (Schwendtner, 2006 ▸), while the X-ray powder diffraction pattern of CsIn(HAsO_4_)_2_ showed a few peaks of an unidentified impurity.

Measured X-ray powder diffraction diagrams of RbIn(HAsO_4_)_2_ and CsIn(HAsO_4_)_2_ were deposited at the Inter­national Centre for Diffraction Data under PDF number 56–1371 (Prem *et al.*, 2005*a* ▸) for RbIn(HAsO_4_)_2_ and 56–1372 (Prem *et al.*, 2005*b* ▸) for CsIn(HAsO_4_)_2_.

The chemical composition of the title compounds was checked by standard SEM–EDX analysis of several crystals of each compound; no impurities could be detected.

## Refinement   

Crystal data, data collection and structure refinement details are summarized in Table 7 ▸.

For all three refinements, the atomic coordinates of the first description of the respective structure types [KSc(HAsO_4_)_2_ (Schwendtner & Kolitsch, 2004*a* ▸) and RbFe(HPO_4_)_2_ (Lii & Wu, 1994 ▸)] were used as initial parameters for better comparison. Hydrogen atoms and additional disordered positions were then located from difference-Fourier maps and added to the respective models.

The two K-atom positions in KIn(HAsO_4_)_2_ were restrained to give a total occupancy of one. Freely refined occupancies were 0.989 (4) (K1) and 0.029 (4) (K2), *i.e.* very close to the ideal bulk occupancy of 1.00. Also the anisotropic displacement parameters were restrained to the same values. The O—H bond lengths were restrained to 0.90 (4) (K compound) and 0.90 (2) Å (Rb and Cs compounds). Residual electron-density peaks of 1.02 and 1.03 e Å^−3^ were encountered close to the Rb1 and Rb2 positions. It seems that the Rb atoms, similarly to what was found for isotypic RbAl(HPO_4_)_2_ (Lesage *et al.*, 2007 ▸), have irregular atomic displacement parameters; therefore, two further, low-occupancy Rb positions, Rb1*B* and Rb2*B*, were included in the refinement to model this positional disorder. The occupancies were accordingly restrained to give a total occupancy of 1.00 for Rb1 and Rb2 [Rb1*a* = 0.949 (3), 3 × Rb1*b* = 0.0170 (9), Rb2*a* = 0.567 (3), 3 × Rb2*b* = 0.1442 (9)]. The refined Rb1*A*—R1*B*, Rb1*B*—R1*B*′, Rb2*A*—R2*B*′ and Rb2*B*—Rb2*B* distances are 0.44 (3), 0.76 (5), 0.249 (8) and 0.423 (14) Å, respectively. The anisotropic displacement parameters of Rb1*a* and Rb1*b*, as well as Rb2*a* and Rb2*b*, were restrained to give the same value.

**Figure 6:**
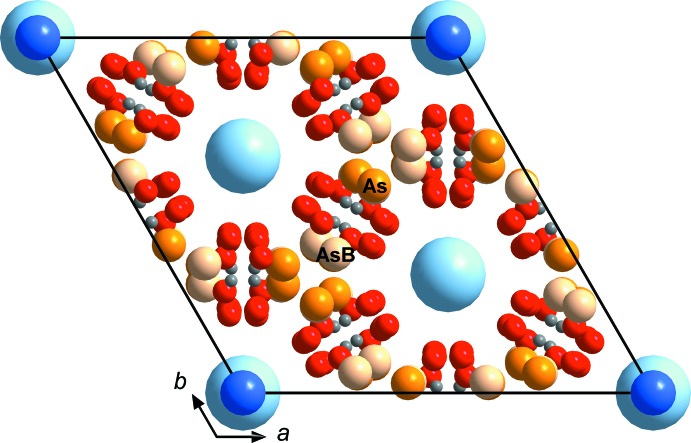
Possible second As position (As*B*) in CsIn(HAsO_4_)_2_, which could explain the residual electron densities. The As*B* position can roughly be generated by a mirror plane in (110). See text for discussion.

The highest residual electron densities are 2.03 e Å^−3^ in CsIn(HAsO_4_)_2_. They are located about 1.65 Å from As at the same *z* coordinate value. At first, it seemed sensible that this position is a ‘flipped’ As position centring an alternative location of the AsO_4_ tetra­hedron. An unrestrained refinement of this position led to occupancy factors of 0.984 (2) for As and 0.015 (2) for the second position and *R*1 decreased from 2.17 to 1.99%. However, the isotropic displacement parameter of the second position refined to zero, which suggested that this position may be an artifact. The position can be generated by a mirror plane in (110) (Fig. 6 ▸). Since application of appropriate twin matrices to the original model did not improve the refinement and since O ligands for this second possible As position could not be detected, the position was omitted from the model.

The highest residual electron densities of RbIn(HAsO_4_)_2_ are at or below 1 e Å^−3^ and 1.43 Å from atom O4. The highest residual electron densities of KIn(HAsO_4_)_2_ are 1.18 e Å^−3^ and close to the As position.

## Supplementary Material

Crystal structure: contains datablock(s) KInHAsO42, RbInHAsO42, CsInHAsO42. DOI: 10.1107/S205698901701355X/pj2047sup1.cif

Structure factors: contains datablock(s) KInHAsO42. DOI: 10.1107/S205698901701355X/pj2047KInHAsO42sup2.hkl

Structure factors: contains datablock(s) RbInHAsO42. DOI: 10.1107/S205698901701355X/pj2047RbInHAsO42sup3.hkl

Structure factors: contains datablock(s) CsInHAsO42. DOI: 10.1107/S205698901701355X/pj2047CsInHAsO42sup4.hkl

CCDC references: 1575921, 1575920, 1575919

Additional supporting information:  crystallographic information; 3D view; checkCIF report

## Figures and Tables

**Table 1:** Selected bond lengths (Å) for KIn(HAsO_4_)

K1—O1^i^	2.6488 (17)	K2—O3^vii^	3.20 (3)
K1—O1	2.6488 (17)	K2—O2	3.33 (3)
K1—O3^ii^	2.7788 (17)	K2—O2^vi^	3.33 (3)
K1—O3^iii^	2.7788 (17)	In—O1	2.1104 (17)
K1—O4^iv^	3.112 (2)	In—O1^vi^	2.1104 (17)
K1—O4^v^	3.112 (2)	In—O3^ii^	2.1388 (16)
K1—O2	3.2553 (19)	In—O3^ix^	2.1388 (16)
K1—O2^i^	3.2553 (19)	In—O2^x^	2.1473 (16)
K2—O4	2.74 (4)	In—O2^v^	2.1473 (16)
K2—O4^vi^	2.74 (4)	As—O1	1.6574 (17)
K2—O1^vii^	2.792 (18)	As—O3	1.6721 (17)
K2—O1^viii^	2.792 (18)	As—O2	1.6762 (16)
K2—O3^viii^	3.20 (3)	As—O4	1.7231 (19)

Symmetry codes: (i) 
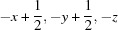
; (ii) 
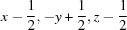
; (iii) 
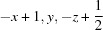
; (iv) 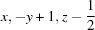; (v) 
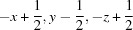
; (vi) 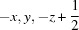; (vii) 
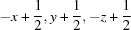
; (viii) 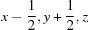; (ix) 
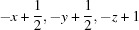
; (x) 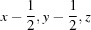.

**Table 2:** Selected bond lengths (Å) for RbIn(HAsO_4_)

Rb1*A*—O3^i^	3.042 (2)	Rb2*A*—O4^xi^	3.668 (5)
Rb1*A*—O3^ii^	3.042 (2)	Rb2*A*—O1^xii^	3.830 (3)
Rb1*A*—O3^iii^	3.042 (2)	Rb2*A*—O1^xiii^	3.830 (3)
Rb1*A*—O3^iv^	3.042 (2)	Rb2*A*—O1^xiv^	3.830 (3)
Rb1*A*—O3^v^	3.042 (2)	In1—O2^xv^	2.1306 (17)
Rb1*A*—O3	3.042 (2)	In1—O2^v^	2.1306 (17)
Rb1*A*—O2	3.3114 (19)	In1—O2^xvi^	2.1306 (17)
Rb1*A*—O2^iv^	3.3115 (19)	In1—O4^xvii^	2.1457 (17)
Rb1*A*—O2^iii^	3.3114 (19)	In1—O4^ii^	2.1457 (16)
Rb1*A*—O2^v^	3.3114 (19)	In1—O4^xii^	2.1457 (16)
Rb1*A*—O2^i^	3.3114 (18)	In2—O1^vii^	2.1312 (19)
Rb1*A*—O2^ii^	3.3114 (18)	In2—O1^xviii^	2.131 (2)
Rb2*A*—O3	3.006 (5)	In2—O1^ii^	2.1312 (19)
Rb2*A*—O3^ii^	3.006 (5)	In2—O1^xii^	2.131 (2)
Rb2*A*—O3^v^	3.006 (5)	In2—O1^xvii^	2.1312 (19)
Rb2*A*—O1^vi^	3.462 (3)	In2—O1^xix^	2.1312 (19)
Rb2*A*—O1^vii^	3.462 (3)	As—O1^xiii^	1.6508 (18)
Rb2*A*—O1^viii^	3.462 (3)	As—O2	1.6668 (17)
Rb2*A*—O4^ix^	3.668 (5)	As—O4^iv^	1.6736 (17)
Rb2*A*—O4^x^	3.668 (5)	As—O3	1.7409 (19)

Symmetry codes: (i) 
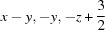
; (ii) 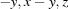; (iii) 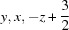; (iv) 
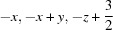
; (v) 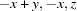; (vi) 
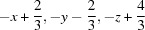
; (vii) 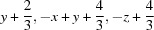; (viii) 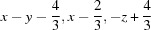; (ix) 
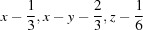
; (x) 
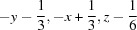
; (xi) 
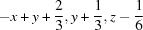
; (xii) 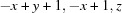; (xiii) 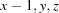; (xiv) 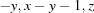; (xv) 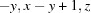; (xvi) 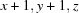; (xvii) 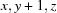; (xviii) 
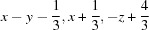
; (xix) 
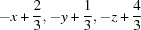
.

**Table 3:** Selected bond lengths (Å) for CsIn(HAsO_4_)

Cs1—O3	3.280 (3)	Cs2—O3^xi^	3.698 (3)
Cs1—O3^i^	3.280 (3)	Cs2—O4^xii^	3.703 (2)
Cs1—O3^ii^	3.280 (3)	Cs2—O4^xiii^	3.703 (2)
Cs1—O3^iii^	3.280 (3)	Cs2—O4^xiv^	3.703 (2)
Cs1—O3^iv^	3.280 (3)	In1—O2^xv^	2.127 (2)
Cs1—O3^v^	3.280 (3)	In1—O2^iii^	2.127 (2)
Cs1—O2	3.434 (2)	In1—O2^xvi^	2.127 (2)
Cs1—O2^ii^	3.434 (2)	In1—O4^xvii^	2.150 (2)
Cs1—O2^v^	3.434 (2)	In1—O4^iv^	2.150 (2)
Cs1—O2^iii^	3.434 (2)	In1—O4^xviii^	2.150 (2)
Cs1—O2^i^	3.434 (2)	In2—O1^vii^	2.133 (2)
Cs1—O2^iv^	3.434 (2)	In2—O1^xi^	2.133 (3)
Cs2—O3^iv^	3.121 (3)	In2—O1^xix^	2.133 (2)
Cs2—O3^iii^	3.121 (2)	In2—O1^iv^	2.133 (2)
Cs2—O3	3.121 (3)	In2—O1^xviii^	2.133 (3)
Cs2—O1^vi^	3.419 (3)	In2—O1^xvii^	2.133 (2)
Cs2—O1^vii^	3.419 (3)	As—O1^xx^	1.655 (2)
Cs2—O1^viii^	3.419 (3)	As—O2	1.671 (2)
Cs2—O3^ix^	3.698 (3)	As—O4^ii^	1.679 (2)
Cs2—O3^x^	3.698 (3)	As—O3	1.743 (3)

Symmetry codes: (i) 
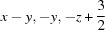
; (ii) 
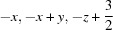
; (iii) 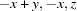; (iv) 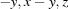; (v) 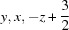; (vi) 
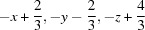
; (vii) 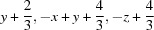; (viii) 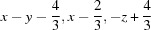; (ix) 
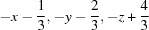
; (x) 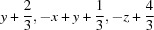; (xi) 
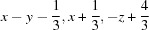
; (xii) 
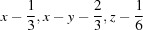
; (xiii) 
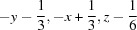
; (xiv) 
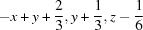
; (xv) 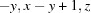; (xvi) 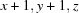; (xvii) 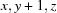; (xviii) 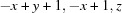; (xix) 
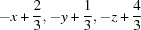
; (xx) 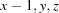.

**Table 4:** Hydrogen-bond geometry (Å, °) for KIn(HAsO_4_)

*D*—H⋯*A*	*D*—H	H⋯*A*	*D*⋯*A*	*D*—H⋯*A*
O4—H⋯O2^xi^	0.88 (2)	1.89 (3)	2.690 (3)	151 (4)

Symmetry code: (xi) 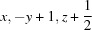.

**Table 5:** Hydrogen-bond geometry (Å, °) for RbIn(HAsO_4_)

*D*—H⋯*A*	*D*—H	H⋯*A*	*D*⋯*A*	*D*—H⋯*A*
O3—H⋯O4^xx^	0.83 (3)	1.82 (3)	2.634 (2)	168 (4)

Symmetry code: (xx) 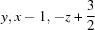.

**Table 6:** Hydrogen-bond geometry (Å, °) for CsIn(HAsO_4_)

*D*—H⋯*A*	*D*—H	H⋯*A*	*D*⋯*A*	*D*—H⋯*A*
O3—H⋯O4^xxi^	0.83 (3)	1.80 (3)	2.621 (3)	170 (4)

Symmetry code: (xxi) 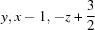.

**Table 7:** Experimental details

	KIn(HAsO_4_)_2_	RbIn(HAsO_4_)_2_	CsIn(HAsO_4_)_2_
Crystal data			
*M*_r_	433.78	480.15	527.59
Crystal system, space group	Monoclinic, *C*2/*c*	Trigonal, *R* 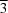 *c*:*H*	Trigonal, *R* 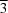 *c*:*H*
Temperature (K)	293	293	293
*a*, *b*, *c* (Å)	8.340 (2), 10.657 (2), 9.197 (2)	8.512 (1), 8.512 (1), 56.434 (11)	8.629 (1), 8.629 (1), 56.986 (11)
α, β, γ (°)	90, 109.37 (3), 90	90, 90, 120	90, 90, 120
*V* (Å^3^)	771.2 (3)	3541.1 (11)	3674.7 (11)
*Z*	4	18	18
Radiation type	Mo *K*α	Mo *K*α	Mo *K*α
μ (mm^−1^)	12.13	17.50	15.34
Crystal size (mm)	0.19 × 0.02 × 0.02	0.05 × 0.05 × 0.02	0.06 × 0.06 × 0.04

Data collection			
Diffractometer	Nonius KappaCCD single-crystal four-circle	Nonius KappaCCD single-crystal four-circle	Nonius KappaCCD single-crystal four-circle
Absorption correction	Multi-scan (*SCALEPACK*; Otwinowski *et al.*, 2003 ▸)	Multi-scan (*SCALEPACK*; Otwinowski *et al.*, 2003 ▸)	Multi-scan (*SCALEPACK*; Otwinowski *et al.*, 2003 ▸)
*T*_min_, *T*_max_	0.207, 0.794	0.475, 0.779	0.460, 0.579
No. of measured, independent and observed [*I* > 2σ(*I*)] reflections	2743, 1406, 1295	5262, 1443, 1255	4350, 1199, 1039
*R*_int_	0.015	0.024	0.019
(sin θ/λ)_max_ (Å^−1^)	0.758	0.757	0.704

Refinement			
*R*[*F* ^2^ > 2σ(*F* ^2^)], *wR*(*F* ^2^), *S*	0.019, 0.046, 1.09	0.018, 0.041, 1.12	0.022, 0.052, 1.07
No. of reflections	1406	1443	1199
No. of parameters	64	69	61
No. of restraints	1	3	1
H-atom treatment	All H-atom parameters refined	All H-atom parameters refined	All H-atom parameters refined
Δρ_max_, Δρ_min_ (e Å^−3^)	1.18, −1.00	1.00, −0.86	2.09, −0.86

Computer programs: *COLLECT* (Nonius, 2003 ▸), *DENZO* and *SCALEPACK* (Otwinowski *et al.*, 2003 ▸), *SHELXS97* (Sheldrick, 2008 ▸), *SHELXL2016* (Sheldrick, 2015 ▸), *DIAMOND* (Brandenburg, 2005 ▸) and *publCIF* (Westrip, 2010 ▸).

## supplementary crystallographic information

### Potassium indium bis[hydrogen arsenate(V)] (KInHAsO42) Crystal data

**Table:**

KIn(HAsO_4_)_2_	*F*(000) = 800
*M**_r_* = 433.78	*D*_x_ = 3.736 Mg m^−^^3^
Monoclinic, *C*2/*c*	Mo *K*α radiation, λ = 0.71073 Å
*a* = 8.340 (2) Å	Cell parameters from 1474 reflections
*b* = 10.657 (2) Å	θ = 3.4–32.6°
*c* = 9.197 (2) Å	µ = 12.13 mm^−^^1^
β = 109.37 (3)°	*T* = 293 K
*V* = 771.2 (3) Å^3^	Small prisms, colourless
*Z* = 4	0.19 × 0.02 × 0.02 mm

### Potassium indium bis[hydrogen arsenate(V)] (KInHAsO42) Data collection

**Table:**

Nonius KappaCCD single-crystal four-circle diffractometer	1295 reflections with *I* > 2σ(*I*)
Radiation source: fine-focus sealed tube	*R*_int_ = 0.015
φ and ω scans	θ_max_ = 32.6°, θ_min_ = 3.4°
Absorption correction: multi-scan (SCALEPACK; Otwinowski *et al.*, 2003)	*h* = −12→12
*T*_min_ = 0.207, *T*_max_ = 0.794	*k* = −16→16
2743 measured reflections	*l* = −13→13
1406 independent reflections	

### Potassium indium bis[hydrogen arsenate(V)] (KInHAsO42) Refinement

**Table:**

Refinement on *F*^2^	Secondary atom site location: difference Fourier map
Least-squares matrix: full	Hydrogen site location: difference Fourier map
*R*[*F*^2^ > 2σ(*F*^2^)] = 0.019	All H-atom parameters refined
*wR*(*F*^2^) = 0.046	*w* = 1/[σ^2^(*F*_o_^2^) + (0.0227*P*)^2^ + 1.2908*P*] where *P* = (*F*_o_^2^ + 2*F*_c_^2^)/3
*S* = 1.09	(Δ/σ)_max_ = 0.001
1406 reflections	Δρ_max_ = 1.18 e Å^−^^3^
64 parameters	Δρ_min_ = −1.00 e Å^−^^3^
1 restraint	Extinction correction: SHELXL2016 (Sheldrick, 2015), Fc^*^=kFc[1+0.001xFc^2^λ^3^/sin(2θ)]^-1/4^
Primary atom site location: structure-invariant direct methods	Extinction coefficient: 0.00181 (17)

### Potassium indium bis[hydrogen arsenate(V)] (KInHAsO42) Special details

**Table:**

Geometry. All esds (except the esd in the dihedral angle between two l.s. planes) are estimated using the full covariance matrix. The cell esds are taken into account individually in the estimation of esds in distances, angles and torsion angles; correlations between esds in cell parameters are only used when they are defined by crystal symmetry. An approximate (isotropic) treatment of cell esds is used for estimating esds involving l.s. planes.

### Potassium indium bis[hydrogen arsenate(V)] (KInHAsO42) Fractional atomic coordinates and isotropic or equivalent isotropic displacement parameters (Å^2^)

**Table:**

	*x*	*y*	*z*	*U*_iso_*/*U*_eq_	Occ. (<1)
K1	0.250000	0.250000	0.000000	0.0437 (3)	0.976 (2)
K2	0.000000	0.678 (5)	0.250000	0.0437 (3)	0.024 (2)
In	0.000000	0.13427 (2)	0.250000	0.00852 (7)	
As	0.27607 (3)	0.39725 (2)	0.36013 (2)	0.00952 (7)	
O1	0.1914 (2)	0.26806 (16)	0.26523 (19)	0.0186 (3)	
O2	0.3114 (2)	0.49181 (17)	0.22827 (18)	0.0177 (3)	
O3	0.4522 (2)	0.36289 (15)	0.50672 (18)	0.0139 (3)	
O4	0.1329 (2)	0.4729 (2)	0.4285 (2)	0.0254 (4)	
H	0.159 (6)	0.473 (5)	0.529 (2)	0.076 (16)*	

### Potassium indium bis[hydrogen arsenate(V)] (KInHAsO42) Atomic displacement parameters (Å^2^)

**Table:**

	*U*^11^	*U*^22^	*U*^33^	*U*^12^	*U*^13^	*U*^23^
K1	0.0550 (7)	0.0588 (7)	0.0301 (5)	−0.0355 (6)	0.0312 (5)	−0.0195 (5)
K2	0.0550 (7)	0.0588 (7)	0.0301 (5)	−0.0355 (6)	0.0312 (5)	−0.0195 (5)
In	0.00910 (10)	0.00802 (11)	0.00799 (10)	0.000	0.00224 (7)	0.000
As	0.01099 (11)	0.00995 (12)	0.00731 (10)	−0.00206 (7)	0.00262 (8)	−0.00008 (7)
O1	0.0243 (9)	0.0157 (8)	0.0181 (8)	−0.0108 (7)	0.0103 (7)	−0.0055 (6)
O2	0.0199 (8)	0.0178 (8)	0.0114 (7)	−0.0096 (6)	−0.0003 (6)	0.0062 (6)
O3	0.0138 (7)	0.0176 (8)	0.0087 (7)	0.0024 (6)	0.0015 (6)	−0.0005 (5)
O4	0.0192 (9)	0.0402 (12)	0.0152 (8)	0.0122 (8)	0.0035 (7)	−0.0053 (8)

### Potassium indium bis[hydrogen arsenate(V)] (KInHAsO42) Geometric parameters (Å, º)

**Table:**

K1—O1^i^	2.6488 (17)	K2—O3^ix^	3.20 (3)
K1—O1	2.6488 (17)	K2—O2	3.33 (3)
K1—K2^ii^	2.653 (15)	K2—O2^viii^	3.33 (3)
K1—K2^iii^	2.653 (15)	K2—As^ix^	3.35 (4)
K1—O3^iv^	2.7788 (17)	K2—As^x^	3.35 (4)
K1—O3^v^	2.7788 (17)	In—O1	2.1104 (17)
K1—O4^vi^	3.112 (2)	In—O1^viii^	2.1104 (17)
K1—O4^vii^	3.112 (2)	In—O3^iv^	2.1388 (16)
K1—O2	3.2553 (19)	In—O3^xi^	2.1388 (16)
K1—O2^i^	3.2553 (19)	In—O2^xii^	2.1473 (16)
K1—As	3.6059 (7)	In—O2^vii^	2.1473 (16)
K1—As^i^	3.6059 (7)	As—O1	1.6574 (17)
K2—O4	2.74 (4)	As—O3	1.6721 (17)
K2—O4^viii^	2.74 (4)	As—O2	1.6762 (16)
K2—O1^ix^	2.792 (18)	As—O4	1.7231 (19)
K2—O1^x^	2.792 (18)	O4—H	0.876 (19)
K2—O3^x^	3.20 (3)		
			
O1^i^—K1—O1	180.0	K1^ix^—K2—As^ix^	72.8 (8)
O1^i^—K1—K2^ii^	63.6 (3)	K1^x^—K2—As^ix^	83.9 (10)
O1—K1—K2^ii^	116.4 (3)	O4—K2—As^ix^	125.63 (13)
O1^i^—K1—K2^iii^	116.4 (3)	O4^viii^—K2—As^ix^	122.06 (11)
O1—K1—K2^iii^	63.6 (3)	O1^ix^—K2—As^ix^	29.6 (4)
K2^ii^—K1—K2^iii^	180 (2)	O1^x^—K2—As^ix^	113.1 (16)
O1^i^—K1—O3^iv^	115.35 (5)	O3^x^—K2—As^ix^	90.8 (12)
O1—K1—O3^iv^	64.65 (5)	O3^ix^—K2—As^ix^	29.5 (3)
K2^ii^—K1—O3^iv^	72.1 (6)	O2—K2—As^ix^	82.73 (9)
K2^iii^—K1—O3^iv^	107.9 (6)	O2^viii^—K2—As^ix^	163.5 (6)
O1^i^—K1—O3^v^	64.65 (5)	K1^ix^—K2—As^x^	83.9 (10)
O1—K1—O3^v^	115.35 (5)	K1^x^—K2—As^x^	72.8 (8)
K2^ii^—K1—O3^v^	107.9 (6)	O4—K2—As^x^	122.06 (11)
K2^iii^—K1—O3^v^	72.1 (6)	O4^viii^—K2—As^x^	125.63 (13)
O3^iv^—K1—O3^v^	180.00 (6)	O1^ix^—K2—As^x^	113.1 (16)
O1^i^—K1—O4^vi^	90.87 (5)	O1^x^—K2—As^x^	29.6 (4)
O1—K1—O4^vi^	89.13 (5)	O3^x^—K2—As^x^	29.5 (3)
K2^ii^—K1—O4^vi^	56.1 (11)	O3^ix^—K2—As^x^	90.8 (12)
K2^iii^—K1—O4^vi^	123.9 (11)	O2—K2—As^x^	163.5 (6)
O3^iv^—K1—O4^vi^	101.27 (5)	O2^viii^—K2—As^x^	82.73 (9)
O3^v^—K1—O4^vi^	78.73 (5)	As^ix^—K2—As^x^	91.7 (13)
O1^i^—K1—O4^vii^	89.13 (5)	O1—In—O1^viii^	94.99 (10)
O1—K1—O4^vii^	90.87 (5)	O1—In—O3^iv^	86.23 (7)
K2^ii^—K1—O4^vii^	123.9 (11)	O1^viii^—In—O3^iv^	92.67 (7)
K2^iii^—K1—O4^vii^	56.1 (11)	O1—In—O3^xi^	92.67 (7)
O3^iv^—K1—O4^vii^	78.73 (5)	O1^viii^—In—O3^xi^	86.23 (7)
O3^v^—K1—O4^vii^	101.27 (5)	O3^iv^—In—O3^xi^	178.38 (9)
O4^vi^—K1—O4^vii^	180.0	O1—In—O2^xii^	177.02 (7)
O1^i^—K1—O2	127.87 (5)	O1^viii^—In—O2^xii^	87.52 (7)
O1—K1—O2	52.13 (5)	O3^iv^—In—O2^xii^	92.07 (6)
K2^ii^—K1—O2	104.0 (10)	O3^xi^—In—O2^xii^	89.08 (7)
K2^iii^—K1—O2	76.0 (10)	O1—In—O2^vii^	87.52 (7)
O3^iv^—K1—O2	106.19 (5)	O1^viii^—In—O2^vii^	177.02 (7)
O3^v^—K1—O2	73.81 (5)	O3^iv^—In—O2^vii^	89.08 (7)
O4^vi^—K1—O2	49.92 (5)	O3^xi^—In—O2^vii^	92.07 (6)
O4^vii^—K1—O2	130.08 (5)	O2^xii^—In—O2^vii^	90.01 (10)
O1^i^—K1—O2^i^	52.13 (5)	O1—In—K1^viii^	107.25 (5)
O1—K1—O2^i^	127.87 (5)	O1^viii^—In—K1^viii^	42.55 (5)
K2^ii^—K1—O2^i^	76.0 (10)	O3^iv^—In—K1^viii^	132.97 (5)
K2^iii^—K1—O2^i^	104.0 (10)	O3^xi^—In—K1^viii^	46.31 (5)
O3^iv^—K1—O2^i^	73.80 (5)	O2^xii^—In—K1^viii^	75.69 (5)
O3^v^—K1—O2^i^	106.20 (5)	O2^vii^—In—K1^viii^	135.06 (5)
O4^vi^—K1—O2^i^	130.08 (5)	O1—In—K1	42.55 (5)
O4^vii^—K1—O2^i^	49.92 (5)	O1^viii^—In—K1	107.25 (5)
O2—K1—O2^i^	180.0	O3^iv^—In—K1	46.31 (5)
O1^i^—K1—As	154.71 (4)	O3^xi^—In—K1	132.97 (5)
O1—K1—As	25.29 (4)	O2^xii^—In—K1	135.06 (5)
K2^ii^—K1—As	117.5 (7)	O2^vii^—In—K1	75.69 (5)
K2^iii^—K1—As	62.5 (7)	K1^viii^—In—K1	141.977 (12)
O3^iv^—K1—As	87.14 (4)	O1—In—K2^iii^	36.3 (7)
O3^v^—K1—As	92.86 (4)	O1^viii^—In—K2^iii^	130.9 (7)
O4^vi^—K1—As	72.55 (4)	O3^iv^—In—K2^iii^	80.73 (5)
O4^vii^—K1—As	107.45 (4)	O3^xi^—In—K2^iii^	99.09 (6)
O2—K1—As	27.68 (3)	O2^xii^—In—K2^iii^	140.9 (7)
O2^i^—K1—As	152.32 (3)	O2^vii^—In—K2^iii^	51.8 (7)
O1^i^—K1—As^i^	25.29 (4)	K1^viii^—In—K2^iii^	135.3 (4)
O1—K1—As^i^	154.71 (4)	K1—In—K2^iii^	38.4 (3)
K2^ii^—K1—As^i^	62.5 (7)	O1—In—K2^xii^	130.9 (7)
K2^iii^—K1—As^i^	117.5 (7)	O1^viii^—In—K2^xii^	36.3 (7)
O3^iv^—K1—As^i^	92.86 (4)	O3^iv^—In—K2^xii^	99.09 (6)
O3^v^—K1—As^i^	87.14 (4)	O3^xi^—In—K2^xii^	80.73 (5)
O4^vi^—K1—As^i^	107.45 (4)	O2^xii^—In—K2^xii^	51.8 (7)
O4^vii^—K1—As^i^	72.55 (4)	O2^vii^—In—K2^xii^	140.9 (7)
O2—K1—As^i^	152.32 (3)	K1^viii^—In—K2^xii^	38.4 (3)
O2^i^—K1—As^i^	27.68 (3)	K1—In—K2^xii^	135.3 (4)
As—K1—As^i^	180.0	K2^iii^—In—K2^xii^	167.1 (14)
K1^ix^—K2—K1^x^	147 (2)	O1—As—O3	110.45 (9)
K1^ix^—K2—O4	70.4 (4)	O1—As—O2	105.35 (8)
K1^x^—K2—O4	142.6 (17)	O3—As—O2	113.38 (8)
K1^ix^—K2—O4^viii^	142.6 (17)	O1—As—O4	110.80 (10)
K1^x^—K2—O4^viii^	70.4 (4)	O3—As—O4	109.76 (8)
O4—K2—O4^viii^	73.9 (13)	O2—As—O4	106.99 (10)
K1^ix^—K2—O1^ix^	58.2 (4)	O1—As—K2^iii^	56.3 (5)
K1^x^—K2—O1^ix^	109.3 (10)	O3—As—K2^iii^	70.26 (6)
O4—K2—O1^ix^	96.2 (5)	O2—As—K2^iii^	87.2 (6)
O4^viii^—K2—O1^ix^	116.1 (8)	O4—As—K2^iii^	163.7 (7)
K1^ix^—K2—O1^x^	109.3 (10)	O1—As—K1	43.06 (6)
K1^x^—K2—O1^x^	58.2 (4)	O3—As—K1	114.49 (6)
O4—K2—O1^x^	116.1 (8)	O2—As—K1	64.44 (6)
O4^viii^—K2—O1^x^	96.2 (5)	O4—As—K1	134.49 (6)
O1^ix^—K2—O1^x^	140 (2)	K2^iii^—As—K1	44.66 (8)
K1^ix^—K2—O3^x^	55.8 (6)	O1—As—K2	114.2 (5)
K1^x^—K2—O3^x^	102.0 (12)	O3—As—K2	134.2 (4)
O4—K2—O3^x^	100.01 (14)	O2—As—K2	63.8 (2)
O4^viii^—K2—O3^x^	144.0 (5)	O4—As—K2	43.88 (15)
O1^ix^—K2—O3^x^	99.8 (12)	K2^iii^—As—K2	146.9 (3)
O1^x^—K2—O3^x^	53.8 (5)	K1—As—K2	104.94 (19)
K1^ix^—K2—O3^ix^	102.0 (12)	As—O1—In	140.97 (10)
K1^x^—K2—O3^ix^	55.8 (6)	As—O1—K1	111.65 (8)
O4—K2—O3^ix^	144.0 (5)	In—O1—K1	104.85 (7)
O4^viii^—K2—O3^ix^	100.01 (14)	As—O1—K2^iii^	94.1 (9)
O1^ix^—K2—O3^ix^	53.8 (5)	In—O1—K2^iii^	117.1 (10)
O1^x^—K2—O3^ix^	99.8 (12)	K1—O1—K2^iii^	58.29 (14)
O3^x^—K2—O3^ix^	104.1 (15)	As—O2—In^xiii^	131.05 (9)
K1^ix^—K2—O2	79.6 (4)	As—O2—K1	87.88 (7)
K1^x^—K2—O2	121.6 (6)	In^xiii^—O2—K1	124.98 (7)
O4—K2—O2	52.4 (7)	As—O2—K2	89.3 (6)
O4^viii^—K2—O2	69.8 (10)	In^xiii^—O2—K2	97.7 (7)
O1^ix^—K2—O2	56.7 (3)	K1—O2—K2	123.5 (5)
O1^x^—K2—O2	163.3 (17)	As—O3—In^xi^	130.50 (9)
O3^x^—K2—O2	134.62 (5)	As—O3—K1^v^	129.02 (8)
O3^ix^—K2—O2	91.84 (5)	In^xi^—O3—K1^v^	99.88 (6)
K1^ix^—K2—O2^viii^	121.6 (6)	As—O3—K2^iii^	80.2 (3)
K1^x^—K2—O2^viii^	79.6 (4)	In^xi^—O3—K2^iii^	139.1 (7)
O4—K2—O2^viii^	69.8 (10)	K1^v^—O3—K2^iii^	52.13 (8)
O4^viii^—K2—O2^viii^	52.4 (7)	As—O4—K2	110.3 (2)
O1^ix^—K2—O2^viii^	163.3 (17)	As—O4—K1^ix^	108.30 (10)
O1^x^—K2—O2^viii^	56.7 (3)	K2—O4—K1^ix^	53.4 (7)
O3^x^—K2—O2^viii^	91.84 (5)	As—O4—H	115 (3)
O3^ix^—K2—O2^viii^	134.62 (5)	K2—O4—H	123 (3)
O2—K2—O2^viii^	106.6 (15)	K1^ix^—O4—H	80 (3)

Symmetry codes: (i) −*x*+1/2, −*y*+1/2, −*z*; (ii) −*x*, −*y*+1, −*z*; (iii) *x*+1/2, *y*−1/2, *z*; (iv) *x*−1/2, −*y*+1/2, *z*−1/2; (v) −*x*+1, *y*, −*z*+1/2; (vi) *x*, −*y*+1, *z*−1/2; (vii) −*x*+1/2, *y*−1/2, −*z*+1/2; (viii) −*x*, *y*, −*z*+1/2; (ix) −*x*+1/2, *y*+1/2, −*z*+1/2; (x) *x*−1/2, *y*+1/2, *z*; (xi) −*x*+1/2, −*y*+1/2, −*z*+1; (xii) *x*−1/2, *y*−1/2, *z*; (xiii) *x*+1/2, *y*+1/2, *z*.

### Potassium indium bis[hydrogen arsenate(V)] (KInHAsO42) Hydrogen-bond geometry (Å, º)

**Table:**

*D*—H···*A*	*D*—H	H···*A*	*D*···*A*	*D*—H···*A*
O4—H···O2^xiv^	0.88 (2)	1.89 (3)	2.690 (3)	151 (4)

Symmetry code: (xiv) *x*, −*y*+1, *z*+1/2.

### Rubidium indium bis[hydrogen arsenate(V)] (RbInHAsO42) Crystal data

**Table:**

RbIn(HAsO_4_)_2_	*D*_x_ = 4.053 Mg m^−^^3^
*M**_r_* = 480.15	Mo *K*α radiation, λ = 0.71073 Å
Trigonal, *R*3*c*:*H*	Cell parameters from 2882 reflections
*a* = 8.512 (1) Å	θ = 2.9–32.6°
*c* = 56.434 (11) Å	µ = 17.50 mm^−^^1^
*V* = 3541.1 (11) Å^3^	*T* = 293 K
*Z* = 18	Small hexagonal platelets, colourless
*F*(000) = 3924	0.05 × 0.05 × 0.02 mm

### Rubidium indium bis[hydrogen arsenate(V)] (RbInHAsO42) Data collection

**Table:**

Nonius KappaCCD single-crystal four-circle diffractometer	1255 reflections with *I* > 2σ(*I*)
Radiation source: fine-focus sealed tube	*R*_int_ = 0.024
φ and ω scans	θ_max_ = 32.6°, θ_min_ = 2.9°
Absorption correction: multi-scan (SCALEPACK; Otwinowski *et al.*, 2003)	*h* = −12→12
*T*_min_ = 0.475, *T*_max_ = 0.779	*k* = −10→10
5262 measured reflections	*l* = −85→85
1443 independent reflections	

### Rubidium indium bis[hydrogen arsenate(V)] (RbInHAsO42) Refinement

**Table:**

Refinement on *F*^2^	Secondary atom site location: difference Fourier map
Least-squares matrix: full	Hydrogen site location: difference Fourier map
*R*[*F*^2^ > 2σ(*F*^2^)] = 0.018	All H-atom parameters refined
*wR*(*F*^2^) = 0.041	*w* = 1/[σ^2^(*F*_o_^2^) + (0.014*P*)^2^ + 16.3085*P*] where *P* = (*F*_o_^2^ + 2*F*_c_^2^)/3
*S* = 1.12	(Δ/σ)_max_ = 0.006
1443 reflections	Δρ_max_ = 1.00 e Å^−^^3^
69 parameters	Δρ_min_ = −0.86 e Å^−^^3^
3 restraints	Extinction correction: SHELXL2016 (Sheldrick, 2015), Fc^*^=kFc[1+0.001xFc^2^λ^3^/sin(2θ)]^-1/4^
Primary atom site location: structure-invariant direct methods	Extinction coefficient: 0.000313 (12)

### Rubidium indium bis[hydrogen arsenate(V)] (RbInHAsO42) Special details

**Table:**

Geometry. All esds (except the esd in the dihedral angle between two l.s. planes) are estimated using the full covariance matrix. The cell esds are taken into account individually in the estimation of esds in distances, angles and torsion angles; correlations between esds in cell parameters are only used when they are defined by crystal symmetry. An approximate (isotropic) treatment of cell esds is used for estimating esds involving l.s. planes.

### Rubidium indium bis[hydrogen arsenate(V)] (RbInHAsO42) Fractional atomic coordinates and isotropic or equivalent isotropic displacement parameters (Å^2^)

**Table:**

	*x*	*y*	*z*	*U*_iso_*/*U*_eq_	Occ. (<1)
Rb1A	0.000000	0.000000	0.750000	0.0236 (2)	0.949 (3)
Rb1B	0.000000	−0.051 (4)	0.750000	0.0236 (2)	0.0170 (9)
Rb2A	0.000000	0.000000	0.66882 (10)	0.0423 (6)	0.567 (3)
Rb2B	−0.0304 (11)	−0.0266 (11)	0.66795 (15)	0.0423 (6)	0.1442 (9)
In1	0.333333	0.666667	0.75297 (2)	0.01028 (7)	
In2	0.333333	0.666667	0.666667	0.01217 (8)	
As	−0.39741 (3)	−0.36940 (3)	0.71279 (2)	0.01097 (7)	
O1	0.5235 (3)	−0.3776 (3)	0.68584 (3)	0.0298 (5)	
O2	−0.4271 (2)	−0.2389 (2)	0.73221 (3)	0.0171 (3)	
O3	−0.1635 (2)	−0.2654 (3)	0.70899 (3)	0.0201 (4)	
O4	0.4679 (2)	−0.1119 (2)	0.77738 (3)	0.0155 (3)	
H	−0.131 (6)	−0.339 (5)	0.7128 (7)	0.051 (13)*	

### Rubidium indium bis[hydrogen arsenate(V)] (RbInHAsO42) Atomic displacement parameters (Å^2^)

**Table:**

	*U*^11^	*U*^22^	*U*^33^	*U*^12^	*U*^13^	*U*^23^
Rb1A	0.0267 (3)	0.0267 (3)	0.0175 (3)	0.01335 (15)	0.000	0.000
Rb1B	0.0267 (3)	0.0267 (3)	0.0175 (3)	0.01335 (15)	0.000	0.000
Rb2A	0.0509 (8)	0.0509 (8)	0.0250 (4)	0.0254 (4)	0.000	0.000
Rb2B	0.0509 (8)	0.0509 (8)	0.0250 (4)	0.0254 (4)	0.000	0.000
In1	0.01052 (8)	0.01052 (8)	0.00981 (12)	0.00526 (4)	0.000	0.000
In2	0.01472 (11)	0.01472 (11)	0.00707 (16)	0.00736 (6)	0.000	0.000
As	0.01290 (11)	0.01207 (11)	0.01030 (10)	0.00802 (9)	0.00056 (8)	0.00086 (8)
O1	0.0399 (12)	0.0513 (14)	0.0129 (8)	0.0337 (12)	−0.0062 (8)	−0.0002 (8)
O2	0.0170 (8)	0.0122 (8)	0.0203 (8)	0.0060 (7)	0.0076 (7)	−0.0012 (6)
O3	0.0145 (8)	0.0163 (9)	0.0308 (10)	0.0087 (7)	0.0061 (7)	0.0072 (7)
O4	0.0137 (8)	0.0133 (8)	0.0217 (8)	0.0084 (6)	−0.0038 (6)	−0.0056 (6)

### Rubidium indium bis[hydrogen arsenate(V)] (RbInHAsO42) Geometric parameters (Å, º)

**Table:**

Rb1A—Rb1B^i^	0.44 (3)	Rb2A—O1^vi^	3.462 (3)
Rb1A—Rb1B^ii^	0.44 (3)	Rb2A—O1^vii^	3.462 (3)
Rb1A—O3^iii^	3.042 (2)	Rb2A—O1^viii^	3.462 (3)
Rb1A—O3^i^	3.042 (2)	Rb2A—O4^ix^	3.668 (5)
Rb1A—O3^iv^	3.042 (2)	Rb2A—O4^x^	3.668 (5)
Rb1A—O3^v^	3.042 (2)	Rb2A—O4^xi^	3.668 (5)
Rb1A—O3^ii^	3.042 (2)	Rb2A—O1^xii^	3.830 (3)
Rb1A—O3	3.042 (2)	Rb2A—O1^xiii^	3.830 (3)
Rb1A—O2	3.3114 (19)	Rb2A—O1^xiv^	3.830 (3)
Rb1A—O2^v^	3.3115 (19)	Rb2A—O3^xv^	3.888 (4)
Rb1A—O2^iv^	3.3114 (19)	Rb2B—Rb2B^i^	0.423 (14)
Rb1A—O2^ii^	3.3114 (19)	Rb2B—Rb2B^ii^	0.423 (14)
Rb1A—O2^iii^	3.3114 (18)	Rb2B—O3	2.911 (9)
Rb1A—O2^i^	3.3114 (18)	Rb2B—O3^ii^	3.056 (9)
Rb1A—As^iii^	3.8862 (5)	Rb2B—O3^i^	3.185 (8)
Rb1A—H	3.28 (4)	Rb2B—O1^viii^	3.259 (8)
Rb1A—H^i^	3.28 (4)	Rb2B—O1^vi^	3.436 (9)
Rb1A—H^ii^	3.28 (4)	Rb2B—O4^x^	3.525 (8)
Rb1A—H^iv^	3.28 (4)	Rb2B—O1^xiii^	3.608 (9)
Rb1A—H^iii^	3.28 (4)	Rb2B—As^xvi^	3.781 (8)
Rb1A—H^v^	3.28 (4)	Rb2B—As^xv^	3.869 (8)
Rb1B—Rb1B^i^	0.76 (6)	Rb2B—As	3.945 (9)
Rb1B—Rb1B^ii^	0.76 (5)	In1—O2^xvii^	2.1306 (17)
Rb1B—O3^v^	2.842 (12)	In1—O2^ii^	2.1306 (17)
Rb1B—O3	2.842 (12)	In1—O2^xviii^	2.1306 (17)
Rb1B—O2^iv^	2.98 (2)	In1—O4^xix^	2.1457 (17)
Rb1B—O2^i^	2.98 (2)	In1—O4^i^	2.1457 (16)
Rb1B—O3^iv^	3.035 (3)	In1—O4^xii^	2.1457 (16)
Rb1B—O3^i^	3.035 (3)	In2—O1^vii^	2.1312 (19)
Rb1B—O2	3.312 (3)	In2—O1^xvi^	2.131 (2)
Rb1B—O2^v^	3.312 (3)	In2—O1^i^	2.1312 (19)
Rb1B—O3^iii^	3.32 (2)	In2—O1^xii^	2.131 (2)
Rb1B—O3^ii^	3.32 (2)	In2—O1^xix^	2.1312 (19)
Rb1B—H	2.99 (4)	In2—O1^xx^	2.1312 (19)
Rb1B—H^v^	2.99 (4)	As—O1^xiii^	1.6508 (18)
Rb2A—Rb2B^ii^	0.249 (8)	As—O2	1.6668 (17)
Rb2A—Rb2B^i^	0.249 (8)	As—O4^v^	1.6736 (17)
Rb2A—O3	3.006 (5)	As—O3	1.7409 (19)
Rb2A—O3^i^	3.006 (5)	O3—H	0.83 (3)
Rb2A—O3^ii^	3.006 (5)		
			
Rb1B^i^—Rb1A—Rb1B^ii^	120.00 (12)	O3—Rb2A—O1^xiv^	76.69 (8)
Rb1B^i^—Rb1A—O3^iii^	59.08 (4)	O3^i^—Rb2A—O1^xiv^	44.11 (5)
Rb1B^ii^—Rb1A—O3^iii^	85.05 (9)	O3^ii^—Rb2A—O1^xiv^	112.37 (15)
Rb1B^i^—Rb1A—O3^i^	59.08 (4)	O1^vi^—Rb2A—O1^xiv^	45.46 (6)
Rb1B^ii^—Rb1A—O3^i^	126.88 (5)	O1^vii^—Rb2A—O1^xiv^	95.29 (5)
O3^iii^—Rb1A—O3^i^	118.16 (8)	O1^viii^—Rb2A—O1^xiv^	150.55 (6)
Rb1B^i^—Rb1A—O3^iv^	126.88 (4)	O4^ix^—Rb2A—O1^xiv^	78.40 (6)
Rb1B^ii^—Rb1A—O3^iv^	59.08 (7)	O4^x^—Rb2A—O1^xiv^	120.81 (10)
O3^iii^—Rb1A—O3^iv^	68.39 (6)	O4^xi^—Rb2A—O1^xiv^	111.19 (9)
O3^i^—Rb1A—O3^iv^	170.10 (7)	O1^xii^—Rb2A—O1^xiv^	113.93 (7)
Rb1B^i^—Rb1A—O3^v^	85.05 (4)	O1^xiii^—Rb2A—O1^xiv^	113.93 (7)
Rb1B^ii^—Rb1A—O3^v^	126.88 (5)	Rb2B^ii^—Rb2A—O3^xv^	126 (3)
O3^iii^—Rb1A—O3^v^	68.39 (6)	Rb2B^i^—Rb2A—O3^xv^	74 (3)
O3^i^—Rb1A—O3^v^	106.24 (7)	O3—Rb2A—O3^xv^	89.86 (5)
O3^iv^—Rb1A—O3^v^	68.39 (6)	O3^i^—Rb2A—O3^xv^	129.48 (6)
Rb1B^i^—Rb1A—O3^ii^	85.05 (4)	O3^ii^—Rb2A—O3^xv^	145.79 (7)
Rb1B^ii^—Rb1A—O3^ii^	59.08 (7)	O1^vi^—Rb2A—O3^xv^	42.24 (6)
O3^iii^—Rb1A—O3^ii^	106.24 (7)	O1^vii^—Rb2A—O3^xv^	117.63 (15)
O3^i^—Rb1A—O3^ii^	68.39 (6)	O1^viii^—Rb2A—O3^xv^	66.65 (8)
O3^iv^—Rb1A—O3^ii^	118.16 (7)	O4^ix^—Rb2A—O3^xv^	42.47 (6)
O3^v^—Rb1A—O3^ii^	170.10 (8)	O4^x^—Rb2A—O3^xv^	40.66 (6)
Rb1B^i^—Rb1A—O3	126.88 (4)	O4^xi^—Rb2A—O3^xv^	76.43 (11)
Rb1B^ii^—Rb1A—O3	85.05 (9)	O1^xii^—Rb2A—O3^xv^	151.71 (14)
O3^iii^—Rb1A—O3	170.10 (7)	O1^xiii^—Rb2A—O3^xv^	69.64 (5)
O3^i^—Rb1A—O3	68.38 (6)	O1^xiv^—Rb2A—O3^xv^	87.30 (5)
O3^iv^—Rb1A—O3	106.24 (7)	Rb2B^i^—Rb2B—Rb2B^ii^	60.00 (6)
O3^v^—Rb1A—O3	118.16 (7)	Rb2B^i^—Rb2B—O3	106.1 (12)
O3^ii^—Rb1A—O3	68.38 (6)	Rb2B^ii^—Rb2B—O3	127.22 (14)
Rb1B^i^—Rb1A—O2	148.87 (3)	Rb2B^i^—Rb2B—O3^ii^	104.1 (12)
Rb1B^ii^—Rb1A—O2	37.75 (11)	Rb2B^ii^—Rb2B—O3^ii^	66.3 (12)
O3^iii^—Rb1A—O2	120.30 (5)	O3—Rb2B—O3^ii^	69.9 (2)
O3^i^—Rb1A—O2	112.03 (5)	Rb2B^i^—Rb2B—O3^i^	46.66 (16)
O3^iv^—Rb1A—O2	67.27 (5)	Rb2B^ii^—Rb2B—O3^i^	68.5 (12)
O3^v^—Rb1A—O2	125.02 (5)	O3—Rb2B—O3^i^	68.05 (19)
O3^ii^—Rb1A—O2	64.77 (5)	O3^ii^—Rb2B—O3^i^	66.39 (17)
O3—Rb1A—O2	50.13 (4)	Rb2B^i^—Rb2B—O1^viii^	158.2 (7)
Rb1B^i^—Rb1A—O2^v^	37.75 (3)	Rb2B^ii^—Rb2B—O1^viii^	111 (2)
Rb1B^ii^—Rb1A—O2^v^	148.87 (10)	O3—Rb2B—O1^viii^	94.9 (2)
O3^iii^—Rb1A—O2^v^	64.77 (5)	O3^ii^—Rb2B—O1^viii^	88.5 (2)
O3^i^—Rb1A—O2^v^	67.27 (5)	O3^i^—Rb2B—O1^viii^	153.0 (3)
O3^iv^—Rb1A—O2^v^	112.03 (5)	Rb2B^i^—Rb2B—O1^vi^	62.0 (19)
O3^v^—Rb1A—O2^v^	50.13 (5)	Rb2B^ii^—Rb2B—O1^vi^	118.2 (19)
O3^ii^—Rb1A—O2^v^	120.30 (5)	O3—Rb2B—O1^vi^	87.5 (2)
O3—Rb1A—O2^v^	125.02 (5)	O3^ii^—Rb2B—O1^vi^	149.7 (3)
O2—Rb1A—O2^v^	172.51 (6)	O3^i^—Rb2B—O1^vi^	86.8 (2)
Rb1B^i^—Rb1A—O2^iv^	148.87 (3)	O1^viii^—Rb2B—O1^vi^	114.2 (2)
Rb1B^ii^—Rb1A—O2^iv^	86.26 (12)	Rb2B^i^—Rb2B—O4^x^	113.2 (3)
O3^iii^—Rb1A—O2^iv^	112.03 (5)	Rb2B^ii^—Rb2B—O4^x^	105.2 (7)
O3^i^—Rb1A—O2^iv^	120.30 (5)	O3—Rb2B—O4^x^	125.4 (3)
O3^iv^—Rb1A—O2^iv^	50.13 (4)	O3^ii^—Rb2B—O4^x^	130.1 (3)
O3^v^—Rb1A—O2^iv^	64.77 (5)	O3^i^—Rb2B—O4^x^	159.7 (3)
O3^ii^—Rb1A—O2^iv^	125.02 (5)	O1^viii^—Rb2B—O4^x^	47.08 (11)
O3—Rb1A—O2^iv^	67.27 (5)	O1^vi^—Rb2B—O4^x^	79.33 (17)
O2—Rb1A—O2^iv^	62.27 (6)	Rb2B^i^—Rb2B—O1^xiii^	150.5 (18)
O2^v^—Rb1A—O2^iv^	111.23 (3)	Rb2B^ii^—Rb2B—O1^xiii^	141.8 (19)
Rb1B^i^—Rb1A—O2^ii^	37.75 (3)	O3—Rb2B—O1^xiii^	47.02 (14)
Rb1B^ii^—Rb1A—O2^ii^	86.26 (12)	O3^ii^—Rb2B—O1^xiii^	79.7 (2)
O3^iii^—Rb1A—O2^ii^	67.27 (5)	O3^i^—Rb2B—O1^xiii^	113.9 (3)
O3^i^—Rb1A—O2^ii^	64.77 (5)	O1^viii^—Rb2B—O1^xiii^	48.49 (13)
O3^iv^—Rb1A—O2^ii^	125.02 (5)	O1^vi^—Rb2B—O1^xiii^	99.9 (2)
O3^v^—Rb1A—O2^ii^	120.30 (5)	O4^x^—Rb2B—O1^xiii^	83.29 (17)
O3^ii^—Rb1A—O2^ii^	50.13 (5)	Rb2B^i^—Rb2B—As^xvi^	132.4 (6)
O3—Rb1A—O2^ii^	112.03 (5)	Rb2B^ii^—Rb2B—As^xvi^	98.8 (15)
O2—Rb1A—O2^ii^	111.23 (3)	O3—Rb2B—As^xvi^	119.4 (2)
O2^v^—Rb1A—O2^ii^	75.50 (6)	O3^ii^—Rb2B—As^xvi^	104.1 (2)
O2^iv^—Rb1A—O2^ii^	172.51 (6)	O3^i^—Rb2B—As^xvi^	166.2 (3)
Rb1B^i^—Rb1A—O2^iii^	86.26 (3)	O1^viii^—Rb2B—As^xvi^	25.78 (7)
Rb1B^ii^—Rb1A—O2^iii^	37.75 (11)	O1^vi^—Rb2B—As^xvi^	104.6 (2)
O3^iii^—Rb1A—O2^iii^	50.13 (4)	O4^x^—Rb2B—As^xvi^	26.18 (6)
O3^i^—Rb1A—O2^iii^	125.02 (5)	O1^xiii^—Rb2B—As^xvi^	72.35 (15)
O3^iv^—Rb1A—O2^iii^	64.77 (5)	Rb2B^i^—Rb2B—As^xv^	75.0 (15)
O3^v^—Rb1A—O2^iii^	112.03 (5)	Rb2B^ii^—Rb2B—As^xv^	117.0 (13)
O3^ii^—Rb1A—O2^iii^	67.27 (5)	O3—Rb2B—As^xv^	105.0 (2)
O3—Rb1A—O2^iii^	120.30 (5)	O3^ii^—Rb2B—As^xv^	174.4 (3)
O2—Rb1A—O2^iii^	75.50 (6)	O3^i^—Rb2B—As^xv^	110.1 (2)
O2^v^—Rb1A—O2^iii^	111.23 (3)	O1^viii^—Rb2B—As^xv^	94.17 (18)
O2^iv^—Rb1A—O2^iii^	111.23 (3)	O1^vi^—Rb2B—As^xv^	25.24 (7)
O2^ii^—Rb1A—O2^iii^	62.27 (6)	O4^x^—Rb2B—As^xv^	54.35 (12)
Rb1B^i^—Rb1A—O2^i^	86.26 (3)	O1^xiii^—Rb2B—As^xv^	98.42 (18)
Rb1B^ii^—Rb1A—O2^i^	148.87 (11)	As^xvi^—Rb2B—As^xv^	80.12 (15)
O3^iii^—Rb1A—O2^i^	125.02 (5)	Rb2B^i^—Rb2B—As	129.9 (12)
O3^i^—Rb1A—O2^i^	50.13 (4)	Rb2B^ii^—Rb2B—As	133.2 (10)
O3^iv^—Rb1A—O2^i^	120.30 (5)	O3—Rb2B—As	23.86 (9)
O3^v^—Rb1A—O2^i^	67.27 (5)	O3^ii^—Rb2B—As	67.16 (17)
O3^ii^—Rb1A—O2^i^	112.03 (5)	O3^i^—Rb2B—As	89.1 (2)
O3—Rb1A—O2^i^	64.77 (5)	O1^viii^—Rb2B—As	71.31 (17)
O2—Rb1A—O2^i^	111.23 (3)	O1^vi^—Rb2B—As	100.0 (2)
O2^v^—Rb1A—O2^i^	62.27 (6)	O4^x^—Rb2B—As	107.7 (2)
O2^iv^—Rb1A—O2^i^	75.50 (6)	O1^xiii^—Rb2B—As	24.73 (7)
O2^ii^—Rb1A—O2^i^	111.23 (3)	As^xvi^—Rb2B—As	96.34 (18)
O2^iii^—Rb1A—O2^i^	172.51 (7)	As^xv^—Rb2B—As	109.1 (2)
Rb1B^i^—Rb1A—As^iii^	68.045 (6)	O2^xvii^—In1—O2^ii^	92.65 (7)
Rb1B^ii^—Rb1A—As^iii^	62.23 (10)	O2^xvii^—In1—O2^xviii^	92.65 (7)
O3^iii^—Rb1A—As^iii^	25.58 (3)	O2^ii^—In1—O2^xviii^	92.65 (7)
O3^i^—Rb1A—As^iii^	121.18 (4)	O2^xvii^—In1—O4^xix^	91.95 (7)
O3^iv^—Rb1A—As^iii^	68.11 (4)	O2^ii^—In1—O4^xix^	173.42 (7)
O3^v^—Rb1A—As^iii^	92.38 (4)	O2^xviii^—In1—O4^xix^	91.82 (7)
O3^ii^—Rb1A—As^iii^	83.92 (4)	O2^xvii^—In1—O4^i^	91.82 (7)
O3—Rb1A—As^iii^	145.24 (3)	O2^ii^—In1—O4^i^	91.95 (7)
O2—Rb1A—As^iii^	99.68 (3)	O2^xviii^—In1—O4^i^	173.42 (7)
O2^v^—Rb1A—As^iii^	86.65 (3)	O4^xix^—In1—O4^i^	83.21 (7)
O2^iv^—Rb1A—As^iii^	118.16 (3)	O2^xvii^—In1—O4^xii^	173.42 (7)
O2^ii^—Rb1A—As^iii^	57.81 (3)	O2^ii^—In1—O4^xii^	91.82 (7)
O2^iii^—Rb1A—As^iii^	25.19 (3)	O2^xviii^—In1—O4^xii^	91.95 (7)
O2^i^—Rb1A—As^iii^	148.88 (3)	O4^xix^—In1—O4^xii^	83.21 (7)
Rb1B^i^—Rb1A—H	127.6 (7)	O4^i^—In1—O4^xii^	83.21 (7)
Rb1B^ii^—Rb1A—H	95.7 (8)	O2^xvii^—In1—Rb2A^xxi^	123.37 (5)
O3^iii^—Rb1A—H	170.2 (6)	O2^ii^—In1—Rb2A^xxi^	123.36 (5)
O3^i^—Rb1A—H	69.0 (7)	O2^xviii^—In1—Rb2A^xxi^	123.36 (5)
O3^iv^—Rb1A—H	103.6 (7)	O4^xix^—In1—Rb2A^xxi^	50.06 (5)
O3^v^—Rb1A—H	103.8 (6)	O4^i^—In1—Rb2A^xxi^	50.06 (5)
O3^ii^—Rb1A—H	82.3 (6)	O4^xii^—In1—Rb2A^xxi^	50.06 (5)
O3—Rb1A—H	14.5 (6)	O2^xvii^—In1—Rb1A^xix^	32.08 (5)
O2—Rb1A—H	58.6 (7)	O2^ii^—In1—Rb1A^xix^	107.92 (5)
O2^v^—Rb1A—H	115.4 (7)	O2^xviii^—In1—Rb1A^xix^	118.90 (5)
O2^iv^—Rb1A—H	58.4 (6)	O4^xix^—In1—Rb1A^xix^	73.96 (4)
O2^ii^—Rb1A—H	122.5 (6)	O4^i^—In1—Rb1A^xix^	63.88 (4)
O2^iii^—Rb1A—H	132.7 (7)	O4^xii^—In1—Rb1A^xix^	141.47 (5)
O2^i^—Rb1A—H	53.3 (7)	Rb2A^xxi^—In1—Rb1A^xix^	91.955 (3)
As^iii^—Rb1A—H	157.8 (7)	O2^xvii^—In1—Rb1A	118.90 (5)
Rb1B^i^—Rb1A—H^i^	44.8 (6)	O2^ii^—In1—Rb1A	32.08 (5)
Rb1B^ii^—Rb1A—H^i^	127.6 (8)	O2^xviii^—In1—Rb1A	107.92 (5)
O3^iii^—Rb1A—H^i^	103.8 (6)	O4^xix^—In1—Rb1A	141.47 (5)
O3^i^—Rb1A—H^i^	14.5 (6)	O4^i^—In1—Rb1A	73.96 (4)
O3^iv^—Rb1A—H^i^	170.2 (6)	O4^xii^—In1—Rb1A	63.88 (5)
O3^v^—Rb1A—H^i^	103.6 (7)	Rb2A^xxi^—In1—Rb1A	91.954 (3)
O3^ii^—Rb1A—H^i^	69.0 (7)	Rb1A^xix^—In1—Rb1A	119.9
O3—Rb1A—H^i^	82.3 (6)	O2^xvii^—In1—Rb1A^xviii^	107.92 (5)
O2—Rb1A—H^i^	122.5 (6)	O2^ii^—In1—Rb1A^xviii^	118.90 (5)
O2^v^—Rb1A—H^i^	58.4 (6)	O2^xviii^—In1—Rb1A^xviii^	32.08 (5)
O2^iv^—Rb1A—H^i^	132.7 (7)	O4^xix^—In1—Rb1A^xviii^	63.88 (5)
O2^ii^—Rb1A—H^i^	53.3 (7)	O4^i^—In1—Rb1A^xviii^	141.47 (5)
O2^iii^—Rb1A—H^i^	115.4 (7)	O4^xii^—In1—Rb1A^xviii^	73.96 (5)
O2^i^—Rb1A—H^i^	58.6 (7)	Rb2A^xxi^—In1—Rb1A^xviii^	91.954 (3)
As^iii^—Rb1A—H^i^	107.6 (7)	Rb1A^xix^—In1—Rb1A^xviii^	119.9
H—Rb1A—H^i^	83.5 (9)	Rb1A—In1—Rb1A^xviii^	119.9
Rb1B^i^—Rb1A—H^ii^	95.7 (7)	O1^vii^—In2—O1^xvi^	96.52 (7)
Rb1B^ii^—Rb1A—H^ii^	44.8 (6)	O1^vii^—In2—O1^i^	180.0
O3^iii^—Rb1A—H^ii^	103.6 (7)	O1^xvi^—In2—O1^i^	83.48 (7)
O3^i^—Rb1A—H^ii^	82.3 (6)	O1^vii^—In2—O1^xii^	83.48 (7)
O3^iv^—Rb1A—H^ii^	103.8 (6)	O1^xvi^—In2—O1^xii^	180.0
O3^v^—Rb1A—H^ii^	170.2 (6)	O1^i^—In2—O1^xii^	96.52 (7)
O3^ii^—Rb1A—H^ii^	14.5 (6)	O1^vii^—In2—O1^xix^	83.48 (7)
O3—Rb1A—H^ii^	69.0 (7)	O1^xvi^—In2—O1^xix^	83.48 (7)
O2—Rb1A—H^ii^	53.3 (7)	O1^i^—In2—O1^xix^	96.52 (7)
O2^v^—Rb1A—H^ii^	132.7 (7)	O1^xii^—In2—O1^xix^	96.52 (7)
O2^iv^—Rb1A—H^ii^	115.4 (8)	O1^vii^—In2—O1^xx^	96.52 (7)
O2^ii^—Rb1A—H^ii^	58.6 (7)	O1^xvi^—In2—O1^xx^	96.52 (7)
O2^iii^—Rb1A—H^ii^	58.4 (6)	O1^i^—In2—O1^xx^	83.48 (7)
O2^i^—Rb1A—H^ii^	122.5 (6)	O1^xii^—In2—O1^xx^	83.48 (7)
As^iii^—Rb1A—H^ii^	78.9 (7)	O1^xix^—In2—O1^xx^	180.0
H—Rb1A—H^ii^	83.5 (9)	O1^vii^—In2—Rb2A^xx^	47.93 (8)
H^i^—Rb1A—H^ii^	83.5 (9)	O1^xvi^—In2—Rb2A^xx^	80.47 (7)
Rb1B^i^—Rb1A—H^iv^	127.6 (7)	O1^i^—In2—Rb2A^xx^	132.07 (8)
Rb1B^ii^—Rb1A—H^iv^	44.8 (6)	O1^xii^—In2—Rb2A^xx^	99.53 (7)
O3^iii^—Rb1A—H^iv^	69.0 (7)	O1^xix^—In2—Rb2A^xx^	37.05 (8)
O3^i^—Rb1A—H^iv^	170.2 (6)	O1^xx^—In2—Rb2A^xx^	142.95 (8)
O3^iv^—Rb1A—H^iv^	14.5 (6)	O1^vii^—In2—Rb2A^xix^	132.07 (8)
O3^v^—Rb1A—H^iv^	82.3 (6)	O1^xvi^—In2—Rb2A^xix^	99.53 (7)
O3^ii^—Rb1A—H^iv^	103.8 (6)	O1^i^—In2—Rb2A^xix^	47.93 (8)
O3—Rb1A—H^iv^	103.6 (7)	O1^xii^—In2—Rb2A^xix^	80.47 (7)
O2—Rb1A—H^iv^	58.4 (6)	O1^xix^—In2—Rb2A^xix^	142.95 (8)
O2^v^—Rb1A—H^iv^	122.5 (6)	O1^xx^—In2—Rb2A^xix^	37.05 (8)
O2^iv^—Rb1A—H^iv^	58.6 (7)	Rb2A^xx^—In2—Rb2A^xix^	180.0
O2^ii^—Rb1A—H^iv^	115.4 (7)	O1^vii^—In2—Rb2A	37.05 (8)
O2^iii^—Rb1A—H^iv^	53.3 (7)	O1^xvi^—In2—Rb2A	132.07 (8)
O2^i^—Rb1A—H^iv^	132.7 (7)	O1^i^—In2—Rb2A	142.95 (8)
As^iii^—Rb1A—H^iv^	62.0 (7)	O1^xii^—In2—Rb2A	47.93 (8)
H—Rb1A—H^iv^	104.8 (15)	O1^xix^—In2—Rb2A	80.47 (7)
H^i^—Rb1A—H^iv^	168.6 (14)	O1^xx^—In2—Rb2A	99.53 (7)
H^ii^—Rb1A—H^iv^	89.6 (11)	Rb2A^xx^—In2—Rb2A	60.060 (6)
Rb1B^i^—Rb1A—H^iii^	44.8 (6)	Rb2A^xix^—In2—Rb2A	119.940 (6)
Rb1B^ii^—Rb1A—H^iii^	95.7 (8)	O1^vii^—In2—Rb2A^xviii^	99.53 (7)
O3^iii^—Rb1A—H^iii^	14.5 (6)	O1^xvi^—In2—Rb2A^xviii^	37.05 (8)
O3^i^—Rb1A—H^iii^	103.8 (6)	O1^i^—In2—Rb2A^xviii^	80.47 (7)
O3^iv^—Rb1A—H^iii^	82.3 (6)	O1^xii^—In2—Rb2A^xviii^	142.95 (8)
O3^v^—Rb1A—H^iii^	69.0 (7)	O1^xix^—In2—Rb2A^xviii^	47.93 (8)
O3^ii^—Rb1A—H^iii^	103.6 (7)	O1^xx^—In2—Rb2A^xviii^	132.07 (8)
O3—Rb1A—H^iii^	170.2 (6)	Rb2A^xx^—In2—Rb2A^xviii^	60.060 (6)
O2—Rb1A—H^iii^	132.7 (7)	Rb2A^xix^—In2—Rb2A^xviii^	119.940 (7)
O2^v^—Rb1A—H^iii^	53.3 (7)	Rb2A—In2—Rb2A^xviii^	119.940 (6)
O2^iv^—Rb1A—H^iii^	122.5 (6)	O1^vii^—In2—Rb2A^xxii^	80.47 (7)
O2^ii^—Rb1A—H^iii^	58.4 (6)	O1^xvi^—In2—Rb2A^xxii^	142.95 (8)
O2^iii^—Rb1A—H^iii^	58.6 (7)	O1^i^—In2—Rb2A^xxii^	99.53 (7)
O2^i^—Rb1A—H^iii^	115.4 (7)	O1^xii^—In2—Rb2A^xxii^	37.05 (8)
As^iii^—Rb1A—H^iii^	33.5 (7)	O1^xix^—In2—Rb2A^xxii^	132.07 (8)
H—Rb1A—H^iii^	168.6 (14)	O1^xx^—In2—Rb2A^xxii^	47.93 (8)
H^i^—Rb1A—H^iii^	89.6 (12)	Rb2A^xx^—In2—Rb2A^xxii^	119.940 (6)
H^ii^—Rb1A—H^iii^	104.8 (15)	Rb2A^xix^—In2—Rb2A^xxii^	60.060 (6)
H^iv^—Rb1A—H^iii^	83.5 (9)	Rb2A—In2—Rb2A^xxii^	60.061 (6)
Rb1B^i^—Rb1A—H^v^	95.7 (7)	Rb2A^xviii^—In2—Rb2A^xxii^	180.0
Rb1B^ii^—Rb1A—H^v^	127.6 (8)	O1^vii^—In2—Rb2A^xxiii^	142.95 (8)
O3^iii^—Rb1A—H^v^	82.3 (6)	O1^xvi^—In2—Rb2A^xxiii^	47.93 (8)
O3^i^—Rb1A—H^v^	103.6 (7)	O1^i^—In2—Rb2A^xxiii^	37.05 (8)
O3^iv^—Rb1A—H^v^	69.0 (7)	O1^xii^—In2—Rb2A^xxiii^	132.07 (8)
O3^v^—Rb1A—H^v^	14.5 (6)	O1^xix^—In2—Rb2A^xxiii^	99.53 (7)
O3^ii^—Rb1A—H^v^	170.2 (6)	O1^xx^—In2—Rb2A^xxiii^	80.47 (7)
O3—Rb1A—H^v^	103.8 (6)	Rb2A^xx^—In2—Rb2A^xxiii^	119.940 (7)
O2—Rb1A—H^v^	115.4 (8)	Rb2A^xix^—In2—Rb2A^xxiii^	60.060 (6)
O2^v^—Rb1A—H^v^	58.6 (7)	Rb2A—In2—Rb2A^xxiii^	180.0
O2^iv^—Rb1A—H^v^	53.3 (7)	Rb2A^xviii^—In2—Rb2A^xxiii^	60.061 (6)
O2^ii^—Rb1A—H^v^	132.7 (7)	Rb2A^xxii^—In2—Rb2A^xxiii^	119.939 (6)
O2^iii^—Rb1A—H^v^	122.5 (6)	O1^xiii^—As—O2	116.08 (10)
O2^i^—Rb1A—H^v^	58.4 (6)	O1^xiii^—As—O4^v^	109.85 (10)
As^iii^—Rb1A—H^v^	105.4 (6)	O2—As—O4^v^	113.73 (8)
H—Rb1A—H^v^	89.6 (11)	O1^xiii^—As—O3	104.27 (10)
H^i^—Rb1A—H^v^	104.8 (14)	O2—As—O3	104.94 (9)
H^ii^—Rb1A—H^v^	168.6 (15)	O4^v^—As—O3	106.99 (8)
H^iv^—Rb1A—H^v^	83.5 (9)	O1^xiii^—As—Rb1B^ii^	133.87 (15)
H^iii^—Rb1A—H^v^	83.5 (9)	O2—As—Rb1B^ii^	51.9 (5)
Rb1B^i^—Rb1B—Rb1B^ii^	60.00 (3)	O4^v^—As—Rb1B^ii^	115.4 (2)
Rb1B^i^—Rb1B—O3^v^	97.5 (5)	O3—As—Rb1B^ii^	54.3 (4)
Rb1B^ii^—Rb1B—O3^v^	123.7 (5)	O1^xiii^—As—Rb1B	138.5 (3)
Rb1B^i^—Rb1B—O3	123.7 (5)	O2—As—Rb1B	62.1 (4)
Rb1B^ii^—Rb1B—O3	97.5 (6)	O4^v^—As—Rb1B	107.4 (4)
O3^v^—Rb1B—O3	133.3 (11)	O3—As—Rb1B	46.49 (19)
Rb1B^i^—Rb1B—O2^iv^	158.5 (3)	Rb1B^ii^—As—Rb1B	11.6 (9)
Rb1B^ii^—Rb1B—O2^iv^	109.8 (5)	O1^xiii^—As—Rb2B^xxiv^	59.16 (16)
O3^v^—Rb1B—O2^iv^	71.7 (5)	O2—As—Rb2B^xxiv^	175.06 (16)
O3—Rb1B—O2^iv^	74.5 (5)	O4^v^—As—Rb2B^xxiv^	68.35 (14)
Rb1B^i^—Rb1B—O2^i^	109.8 (4)	O3—As—Rb2B^xxiv^	78.23 (14)
Rb1B^ii^—Rb1B—O2^i^	158.5 (3)	Rb1B^ii^—As—Rb2B^xxiv^	131.9 (5)
O3^v^—Rb1B—O2^i^	74.5 (5)	Rb1B—As—Rb2B^xxiv^	122.0 (3)
O3—Rb1B—O2^i^	71.7 (5)	O1^xiii^—As—Rb2B^xv^	62.57 (15)
O2^iv^—Rb1B—O2^i^	85.8 (8)	O2—As—Rb2B^xv^	175.80 (17)
Rb1B^i^—Rb1B—O3^iv^	105.8 (5)	O4^v^—As—Rb2B^xv^	70.24 (15)
Rb1B^ii^—Rb1B—O3^iv^	68.2 (6)	O3—As—Rb2B^xv^	72.08 (14)
O3^v^—Rb1B—O3^iv^	71.05 (15)	Rb1B^ii^—As—Rb2B^xv^	125.7 (4)
O3—Rb1B—O3^iv^	111.7 (3)	Rb1B—As—Rb2B^xv^	115.9 (3)
O2^iv^—Rb1B—O3^iv^	53.4 (2)	Rb2B^xxiv^—As—Rb2B^xv^	6.2 (2)
O2^i^—Rb1B—O3^iv^	132.8 (9)	O1^xiii^—As—Rb1A	134.73 (9)
Rb1B^i^—Rb1B—O3^i^	68.2 (5)	O2—As—Rb1A	57.74 (6)
Rb1B^ii^—Rb1B—O3^i^	105.8 (5)	O4^v^—As—Rb1A	112.84 (6)
O3^v^—Rb1B—O3^i^	111.7 (3)	O3—As—Rb1A	48.98 (7)
O3—Rb1B—O3^i^	71.04 (15)	Rb1B^ii^—As—Rb1A	6.0 (4)
O2^iv^—Rb1B—O3^i^	132.8 (9)	Rb1B—As—Rb1A	6.2 (5)
O2^i^—Rb1B—O3^i^	53.4 (2)	Rb2B^xxiv^—As—Rb1A	126.16 (13)
O3^iv^—Rb1B—O3^i^	173.4 (12)	Rb2B^xv^—As—Rb1A	119.97 (12)
Rb1B^i^—Rb1B—O2	114.7 (5)	O1^xiii^—As—Rb2A^xv^	61.31 (9)
Rb1B^ii^—Rb1B—O2	57.8 (6)	O2—As—Rb2A^xv^	177.30 (9)
O3^v^—Rb1B—O2	132.6 (4)	O4^v^—As—Rb2A^xv^	68.43 (8)
O3—Rb1B—O2	51.41 (7)	O3—As—Rb2A^xv^	75.58 (7)
O2^iv^—Rb1B—O2	65.7 (2)	Rb1B^ii^—As—Rb2A^xv^	129.1 (4)
O2^i^—Rb1B—O2	120.6 (6)	Rb1B—As—Rb2A^xv^	119.2 (3)
O3^iv^—Rb1B—O2	67.34 (7)	Rb2B^xxiv^—As—Rb2A^xv^	2.88 (15)
O3^i^—Rb1B—O2	112.19 (11)	Rb2B^xv^—As—Rb2A^xv^	3.52 (13)
Rb1B^i^—Rb1B—O2^v^	57.8 (5)	Rb1A—As—Rb2A^xv^	123.340 (18)
Rb1B^ii^—Rb1B—O2^v^	114.7 (4)	O1^xiii^—As—Rb2B	66.15 (14)
O3^v^—Rb1B—O2^v^	51.41 (7)	O2—As—Rb2B	104.87 (14)
O3—Rb1B—O2^v^	132.6 (4)	O4^v^—As—Rb2B	137.04 (14)
O2^iv^—Rb1B—O2^v^	120.6 (6)	O3—As—Rb2B	42.56 (14)
O2^i^—Rb1B—O2^v^	65.7 (2)	Rb1B^ii^—As—Rb2B	74.7 (2)
O3^iv^—Rb1B—O2^v^	112.19 (11)	Rb1B—As—Rb2B	74.33 (18)
O3^i^—Rb1B—O2^v^	67.34 (7)	Rb2B^xxiv^—As—Rb2B	74.78 (3)
O2—Rb1B—O2^v^	172.4 (11)	Rb2B^xv^—As—Rb2B	70.9 (2)
Rb1B^i^—Rb1B—O3^iii^	45.4 (3)	Rb1A—As—Rb2B	72.61 (11)
Rb1B^ii^—Rb1B—O3^iii^	61.5 (3)	Rb2A^xv^—As—Rb2B	73.66 (14)
O3^v^—Rb1B—O3^iii^	66.84 (19)	O1^xiii^—As—Rb2B^xxv^	63.55 (13)
O3—Rb1B—O3^iii^	158.9 (9)	O2—As—Rb2B^xxv^	178.70 (16)
O2^iv^—Rb1B—O3^iii^	113.43 (5)	O4^v^—As—Rb2B^xxv^	65.54 (13)
O2^i^—Rb1B—O3^iii^	126.87 (6)	O3—As—Rb2B^xxv^	76.36 (14)
O3^iv^—Rb1B—O3^iii^	64.9 (3)	Rb1B^ii^—As—Rb2B^xxv^	129.3 (5)
O3^i^—Rb1B—O3^iii^	110.3 (6)	Rb1B—As—Rb2B^xxv^	119.0 (4)
O2—Rb1B—O3^iii^	112.4 (6)	Rb2B^xxiv^—As—Rb2B^xxv^	4.39 (15)
O2^v^—Rb1B—O3^iii^	61.9 (2)	Rb2B^xv^—As—Rb2B^xxv^	5.30 (19)
Rb1B^i^—Rb1B—O3^ii^	61.5 (3)	Rb1A—As—Rb2B^xxv^	123.48 (12)
Rb1B^ii^—Rb1B—O3^ii^	45.4 (3)	Rb2A^xv^—As—Rb2B^xxv^	2.94 (15)
O3^v^—Rb1B—O3^ii^	158.9 (9)	Rb2B—As—Rb2B^xxv^	76.18 (18)
O3—Rb1B—O3^ii^	66.84 (19)	As^xxvi^—O1—In2^xxvii^	142.20 (12)
O2^iv^—Rb1B—O3^ii^	126.87 (6)	As^xxvi^—O1—Rb2B^xxviii^	95.06 (18)
O2^i^—Rb1B—O3^ii^	113.43 (5)	In2^xxvii^—O1—Rb2B^xxviii^	119.53 (18)
O3^iv^—Rb1B—O3^ii^	110.3 (6)	As^xxvi^—O1—Rb2B^vi^	92.19 (16)
O3^i^—Rb1B—O3^ii^	64.9 (3)	In2^xxvii^—O1—Rb2B^vi^	123.76 (15)
O2—Rb1B—O3^ii^	61.9 (2)	Rb2B^xxviii^—O1—Rb2B^vi^	6.6 (3)
O2^v^—Rb1B—O3^ii^	112.4 (6)	As^xxvi^—O1—Rb2A^vi^	93.97 (12)
O3^iii^—Rb1B—O3^ii^	94.2 (8)	In2^xxvii^—O1—Rb2A^vi^	121.16 (11)
Rb1B^i^—Rb1B—H	135.3 (8)	Rb2B^xxviii^—O1—Rb2A^vi^	2.47 (17)
Rb1B^ii^—Rb1B—H	112.7 (9)	Rb2B^vi^—O1—Rb2A^vi^	4.12 (14)
O3^v^—Rb1B—H	117.3 (12)	As^xxvi^—O1—Rb2B^xxvi^	89.12 (16)
O3—Rb1B—H	16.1 (6)	In2^xxvii^—O1—Rb2B^xxvi^	106.82 (16)
O2^iv^—Rb1B—H	65.2 (9)	Rb2B^xxviii^—O1—Rb2B^xxvi^	86.07 (7)
O2^i^—Rb1B—H	59.4 (9)	Rb2B^vi^—O1—Rb2B^xxvi^	80.1 (2)
O3^iv^—Rb1B—H	111.3 (10)	Rb2A^vi^—O1—Rb2B^xxvi^	83.83 (15)
O3^i^—Rb1B—H	73.1 (8)	As^xxvi^—O1—Rb2A^xxvi^	87.55 (12)
O2—Rb1B—H	61.4 (8)	In2^xxvii^—O1—Rb2A^xxvi^	107.67 (9)
O2^v^—Rb1B—H	124.3 (10)	Rb2B^xxviii^—O1—Rb2A^xxvi^	86.97 (17)
O3^iii^—Rb1B—H	173.7 (9)	Rb2B^vi^—O1—Rb2A^xxvi^	80.92 (17)
O3^ii^—Rb1B—H	82.4 (6)	Rb2A^vi^—O1—Rb2A^xxvi^	84.71 (5)
Rb1B^i^—Rb1B—H^v^	112.7 (9)	Rb2B^xxvi^—O1—Rb2A^xxvi^	1.7 (2)
Rb1B^ii^—Rb1B—H^v^	135.3 (7)	As^xxvi^—O1—Rb2A^xxvii^	110.41 (11)
O3^v^—Rb1B—H^v^	16.1 (6)	In2^xxvii^—O1—Rb2A^xxvii^	74.80 (7)
O3—Rb1B—H^v^	117.3 (12)	Rb2B^xxviii^—O1—Rb2A^xxvii^	65.58 (17)
O2^iv^—Rb1B—H^v^	59.4 (9)	Rb2B^vi^—O1—Rb2A^xxvii^	72.13 (14)
O2^i^—Rb1B—H^v^	65.2 (9)	Rb2A^vi^—O1—Rb2A^xxvii^	68.03 (4)
O3^iv^—Rb1B—H^v^	73.1 (8)	Rb2B^xxvi^—O1—Rb2A^xxvii^	146.21 (11)
O3^i^—Rb1B—H^v^	111.3 (9)	Rb2A^xxvi^—O1—Rb2A^xxvii^	147.76 (12)
O2—Rb1B—H^v^	124.3 (11)	As—O2—In1^xxix^	123.06 (9)
O2^v^—Rb1B—H^v^	61.4 (8)	As—O2—Rb1B^ii^	102.0 (4)
O3^iii^—Rb1B—H^v^	82.4 (6)	In1^xxix^—O2—Rb1B^ii^	124.8 (3)
O3^ii^—Rb1B—H^v^	173.7 (10)	As—O2—Rb1A	97.07 (7)
H—Rb1B—H^v^	101.4 (15)	In1^xxix^—O2—Rb1A	127.94 (7)
Rb2B^ii^—Rb2A—Rb2B^i^	116 (2)	Rb1B^ii^—O2—Rb1A	5.1 (4)
Rb2B^ii^—Rb2A—O3	134 (3)	As—O2—Rb1B	91.5 (4)
Rb2B^i^—Rb2A—O3	99 (3)	In1^xxix^—O2—Rb1B	128.91 (9)
Rb2B^ii^—Rb2A—O3^i^	99 (3)	Rb1B^ii^—O2—Rb1B	12.4 (9)
Rb2B^i^—Rb2A—O3^i^	65 (3)	Rb1A—O2—Rb1B	7.5 (5)
O3—Rb2A—O3^i^	69.31 (13)	As—O2—Rb2A	56.91 (5)
Rb2B^ii^—Rb2A—O3^ii^	65 (3)	In1^xxix^—O2—Rb2A	162.73 (8)
Rb2B^i^—Rb2A—O3^ii^	134 (3)	Rb1B^ii^—O2—Rb2A	68.43 (19)
O3—Rb2A—O3^ii^	69.31 (13)	Rb1A—O2—Rb2A	66.24 (6)
O3^i^—Rb2A—O3^ii^	69.31 (13)	Rb1B—O2—Rb2A	66.61 (7)
Rb2B^ii^—Rb2A—O1^vi^	140 (3)	As—O3—Rb1B	107.13 (15)
Rb2B^i^—Rb2A—O1^vi^	34 (2)	As—O3—Rb2B	113.58 (18)
O3—Rb2A—O1^vi^	85.60 (6)	Rb1B—O3—Rb2B	107.8 (5)
O3^i^—Rb2A—O1^vi^	89.21 (6)	As—O3—Rb2A	117.21 (9)
O3^ii^—Rb2A—O1^vi^	151.25 (17)	Rb1B—O3—Rb2A	103.8 (4)
Rb2B^ii^—Rb2A—O1^vii^	34 (2)	Rb2B—O3—Rb2A	4.46 (18)
Rb2B^i^—Rb2A—O1^vii^	82 (2)	As—O3—Rb1B^ii^	98.0 (5)
O3—Rb2A—O1^vii^	151.25 (17)	Rb1B—O3—Rb1B^ii^	14.3 (10)
O3^i^—Rb2A—O1^vii^	85.60 (6)	Rb2B—O3—Rb1B^ii^	102.66 (17)
O3^ii^—Rb2A—O1^vii^	89.21 (6)	Rb2A—O3—Rb1B^ii^	99.24 (11)
O1^vi^—Rb2A—O1^vii^	108.60 (10)	As—O3—Rb1A	105.44 (8)
Rb2B^ii^—Rb2A—O1^viii^	82 (2)	Rb1B—O3—Rb1A	7.6 (6)
Rb2B^i^—Rb2A—O1^viii^	140 (3)	Rb2B—O3—Rb1A	102.30 (16)
O3—Rb2A—O1^viii^	89.21 (6)	Rb2A—O3—Rb1A	98.50 (9)
O3^i^—Rb2A—O1^viii^	151.25 (17)	Rb1B^ii^—O3—Rb1A	8.2 (6)
O3^ii^—Rb2A—O1^viii^	85.60 (6)	As—O3—Rb2B^i^	121.23 (19)
O1^vi^—Rb2A—O1^viii^	108.60 (10)	Rb1B—O3—Rb2B^i^	103.8 (4)
O1^vii^—Rb2A—O1^viii^	108.59 (10)	Rb2B—O3—Rb2B^i^	7.6 (3)
Rb2B^ii^—Rb2A—O4^ix^	98 (3)	Rb2A—O3—Rb2B^i^	4.62 (15)
Rb2B^i^—Rb2A—O4^ix^	53 (3)	Rb1B^ii^—O3—Rb2B^i^	100.4 (2)
O3—Rb2A—O4^ix^	126.63 (6)	Rb1A—O3—Rb2B^i^	99.01 (18)
O3^i^—Rb2A—O4^ix^	117.83 (6)	As—O3—Rb2B^ii^	115.94 (17)
O3^ii^—Rb2A—O4^ix^	163.52 (11)	Rb1B—O3—Rb2B^ii^	101.7 (5)
O1^vi^—Rb2A—O4^ix^	44.75 (6)	Rb2B—O3—Rb2B^ii^	6.1 (2)
O1^vii^—Rb2A—O4^ix^	77.05 (10)	Rb2A—O3—Rb2B^ii^	3.2 (2)
O1^viii^—Rb2A—O4^ix^	90.14 (12)	Rb1B^ii^—O3—Rb2B^ii^	96.67 (18)
Rb2B^ii^—Rb2A—O4^x^	86 (3)	Rb1A—O3—Rb2B^ii^	96.23 (18)
Rb2B^i^—Rb2A—O4^x^	98 (3)	Rb2B^i^—O3—Rb2B^ii^	7.4 (2)
O3—Rb2A—O4^x^	117.83 (6)	As—O3—Rb1B^i^	110.6 (3)
O3^i^—Rb2A—O4^x^	163.52 (11)	Rb1B—O3—Rb1B^i^	10.9 (8)
O3^ii^—Rb2A—O4^x^	126.63 (6)	Rb2B—O3—Rb1B^i^	97.1 (4)
O1^vi^—Rb2A—O4^x^	77.05 (10)	Rb2A—O3—Rb1B^i^	93.2 (4)
O1^vii^—Rb2A—O4^x^	90.14 (12)	Rb1B^ii^—O3—Rb1B^i^	12.6 (9)
O1^viii^—Rb2A—O4^x^	44.75 (6)	Rb1A—O3—Rb1B^i^	6.0 (4)
O4^ix^—Rb2A—O4^x^	45.71 (8)	Rb2B^i^—O3—Rb1B^i^	93.5 (4)
Rb2B^ii^—Rb2A—O4^xi^	53 (3)	Rb2B^ii^—O3—Rb1B^i^	91.0 (4)
Rb2B^i^—Rb2A—O4^xi^	86 (3)	As—O3—Rb2A^xv^	78.72 (7)
O3—Rb2A—O4^xi^	163.52 (11)	Rb1B—O3—Rb2A^xv^	159.5 (6)
O3^i^—Rb2A—O4^xi^	126.63 (6)	Rb2B—O3—Rb2A^xv^	86.8 (2)
O3^ii^—Rb2A—O4^xi^	117.83 (6)	Rb2A—O3—Rb2A^xv^	90.14 (5)
O1^vi^—Rb2A—O4^xi^	90.14 (12)	Rb1B^ii^—O3—Rb2A^xv^	170.52 (10)
O1^vii^—Rb2A—O4^xi^	44.75 (6)	Rb1A—O3—Rb2A^xv^	167.05 (8)
O1^viii^—Rb2A—O4^xi^	77.05 (10)	Rb2B^i^—O3—Rb2A^xv^	88.8 (2)
O4^ix^—Rb2A—O4^xi^	45.71 (8)	Rb2B^ii^—O3—Rb2A^xv^	92.8 (2)
O4^x^—Rb2A—O4^xi^	45.71 (8)	Rb1B^i^—O3—Rb2A^xv^	167.03 (11)
Rb2B^ii^—Rb2A—O1^xii^	26 (3)	As—O3—H	108 (3)
Rb2B^i^—Rb2A—O1^xii^	117 (2)	Rb1B—O3—H	92 (3)
O3—Rb2A—O1^xii^	112.37 (15)	Rb2B—O3—H	125 (3)
O3^i^—Rb2A—O1^xii^	76.69 (8)	Rb2A—O3—H	124 (3)
O3^ii^—Rb2A—O1^xii^	44.11 (5)	Rb1B^ii^—O3—H	105 (3)
O1^vi^—Rb2A—O1^xii^	150.55 (6)	Rb1A—O3—H	99 (3)
O1^vii^—Rb2A—O1^xii^	45.46 (6)	Rb2B^i^—O3—H	120 (3)
O1^viii^—Rb2A—O1^xii^	95.29 (5)	Rb2B^ii^—O3—H	127 (3)
O4^ix^—Rb2A—O1^xii^	120.81 (10)	Rb1B^i^—O3—H	100 (3)
O4^x^—Rb2A—O1^xii^	111.19 (9)	Rb2A^xv^—O3—H	68 (3)
O4^xi^—Rb2A—O1^xii^	78.40 (6)	As^v^—O4—In1^xxvii^	127.87 (9)
Rb2B^ii^—Rb2A—O1^xiii^	117 (2)	As^v^—O4—Rb2B^xxx^	85.47 (16)
Rb2B^i^—Rb2A—O1^xiii^	126 (2)	In1^xxvii^—O4—Rb2B^xxx^	106.35 (17)
O3—Rb2A—O1^xiii^	44.11 (5)	As^v^—O4—Rb2A^xxx^	86.47 (7)
O3^i^—Rb2A—O1^xiii^	112.37 (15)	In1^xxvii^—O4—Rb2A^xxx^	103.30 (7)
O3^ii^—Rb2A—O1^xiii^	76.69 (8)	Rb2B^xxx^—O4—Rb2A^xxx^	3.25 (18)
O1^vi^—Rb2A—O1^xiii^	95.29 (5)	As^v^—O4—Rb1A^xxvi^	131.05 (7)
O1^vii^—Rb2A—O1^xiii^	150.54 (6)	In1^xxvii^—O4—Rb1A^xxvi^	90.25 (5)
O1^viii^—Rb2A—O1^xiii^	45.46 (6)	Rb2B^xxx^—O4—Rb1A^xxvi^	115.02 (15)
O4^ix^—Rb2A—O1^xiii^	111.19 (9)	Rb2A^xxx^—O4—Rb1A^xxvi^	116.54 (4)
O4^x^—Rb2A—O1^xiii^	78.40 (6)	As^v^—O4—Rb1A	48.38 (5)
O4^xi^—Rb2A—O1^xiii^	120.81 (10)	In1^xxvii^—O4—Rb1A	80.54 (5)
O1^xii^—Rb2A—O1^xiii^	113.93 (7)	Rb2B^xxx^—O4—Rb1A	109.65 (15)
Rb2B^ii^—Rb2A—O1^xiv^	126 (2)	Rb2A^xxx^—O4—Rb1A	108.26 (4)
Rb2B^i^—Rb2A—O1^xiv^	26 (3)	Rb1A^xxvi^—O4—Rb1A	135.18 (4)

Symmetry codes: (i) −*y*, *x*−*y*, *z*; (ii) −*x*+*y*, −*x*, *z*; (iii) *x*−*y*, −*y*, −*z*+3/2; (iv) *y*, *x*, −*z*+3/2; (v) −*x*, −*x*+*y*, −*z*+3/2; (vi) −*x*+2/3, −*y*−2/3, −*z*+4/3; (vii) *y*+2/3, −*x*+*y*+4/3, −*z*+4/3; (viii) *x*−*y*−4/3, *x*−2/3, −*z*+4/3; (ix) *x*−1/3, *x*−*y*−2/3, *z*−1/6; (x) −*y*−1/3, −*x*+1/3, *z*−1/6; (xi) −*x*+*y*+2/3, *y*+1/3, *z*−1/6; (xii) −*x*+*y*+1, −*x*+1, *z*; (xiii) *x*−1, *y*, *z*; (xiv) −*y*, *x*−*y*−1, *z*; (xv) −*x*−1/3, −*y*−2/3, −*z*+4/3; (xvi) *x*−*y*−1/3, *x*+1/3, −*z*+4/3; (xvii) −*y*, *x*−*y*+1, *z*; (xviii) *x*+1, *y*+1, *z*; (xix) *x*, *y*+1, *z*; (xx) −*x*+2/3, −*y*+1/3, −*z*+4/3; (xxi) −*y*+1/3, −*x*+2/3, *z*+1/6; (xxii) −*x*−1/3, −*y*+1/3, −*z*+4/3; (xxiii) −*x*+2/3, −*y*+4/3, −*z*+4/3; (xxiv) *y*−1/3, −*x*+*y*−2/3, −*z*+4/3; (xxv) *x*−*y*−1/3, *x*−2/3, −*z*+4/3; (xxvi) *x*+1, *y*, *z*; (xxvii) *x*, *y*−1, *z*; (xxviii) *y*+2/3, −*x*+*y*−2/3, −*z*+4/3; (xxix) *x*−1, *y*−1, *z*; (xxx) −*y*+1/3, −*x*−1/3, *z*+1/6.

### Rubidium indium bis[hydrogen arsenate(V)] (RbInHAsO42) Hydrogen-bond geometry (Å, º)

**Table:**

*D*—H···*A*	*D*—H	H···*A*	*D*···*A*	*D*—H···*A*
O3—H···O4^xxxi^	0.83 (3)	1.82 (3)	2.634 (2)	168 (4)

Symmetry code: (xxxi) *y*, *x*−1, −*z*+3/2.

### Caesium indium bis[hydrogen arsenate(V)] (CsInHAsO42) Crystal data

**Table:**

CsIn(HAsO_4_)_2_	*D*_x_ = 4.291 Mg m^−^^3^
*M**_r_* = 527.59	Mo *K*α radiation, λ = 0.71073 Å
Trigonal, *R*3*c*:*H*	Cell parameters from 2985 reflections
*a* = 8.629 (1) Å	θ = 3.1–30.0°
*c* = 56.986 (11) Å	µ = 15.34 mm^−^^1^
*V* = 3674.7 (11) Å^3^	*T* = 293 K
*Z* = 18	Small pseudooctahedra, colourless
*F*(000) = 4248	0.06 × 0.06 × 0.04 mm

### Caesium indium bis[hydrogen arsenate(V)] (CsInHAsO42) Data collection

**Table:**

Nonius KappaCCD single-crystal four-circle diffractometer	1039 reflections with *I* > 2σ(*I*)
Radiation source: fine-focus sealed tube	*R*_int_ = 0.019
φ and ω scans	θ_max_ = 30.0°, θ_min_ = 3.1°
Absorption correction: multi-scan (SCALEPACK; Otwinowski *et al.*, 2003)	*h* = −12→12
*T*_min_ = 0.460, *T*_max_ = 0.579	*k* = −9→9
4350 measured reflections	*l* = −79→79
1199 independent reflections	

### Caesium indium bis[hydrogen arsenate(V)] (CsInHAsO42) Refinement

**Table:**

Refinement on *F*^2^	Secondary atom site location: difference Fourier map
Least-squares matrix: full	Hydrogen site location: difference Fourier map
*R*[*F*^2^ > 2σ(*F*^2^)] = 0.022	All H-atom parameters refined
*wR*(*F*^2^) = 0.052	*w* = 1/[σ^2^(*F*_o_^2^) + (0.0234*P*)^2^ + 28.1228*P*] where *P* = (*F*_o_^2^ + 2*F*_c_^2^)/3
*S* = 1.07	(Δ/σ)_max_ = 0.004
1199 reflections	Δρ_max_ = 2.09 e Å^−^^3^
61 parameters	Δρ_min_ = −0.86 e Å^−^^3^
1 restraint	Extinction correction: SHELXL2016 (Sheldrick, 2015), Fc^*^=kFc[1+0.001xFc^2^λ^3^/sin(2θ)]^-1/4^
Primary atom site location: structure-invariant direct methods	Extinction coefficient: 0.000028 (7)

### Caesium indium bis[hydrogen arsenate(V)] (CsInHAsO42) Special details

**Table:**

Geometry. All esds (except the esd in the dihedral angle between two l.s. planes) are estimated using the full covariance matrix. The cell esds are taken into account individually in the estimation of esds in distances, angles and torsion angles; correlations between esds in cell parameters are only used when they are defined by crystal symmetry. An approximate (isotropic) treatment of cell esds is used for estimating esds involving l.s. planes.

### Caesium indium bis[hydrogen arsenate(V)] (CsInHAsO42) Fractional atomic coordinates and isotropic or equivalent isotropic displacement parameters (Å^2^)

**Table:**

	*x*	*y*	*z*	*U*_iso_*/*U*_eq_	
Cs1	0.000000	0.000000	0.750000	0.02557 (14)	
Cs2	0.000000	0.000000	0.66740 (2)	0.02940 (12)	
In1	0.333333	0.666667	0.75230 (2)	0.00967 (10)	
In2	0.333333	0.666667	0.666667	0.01033 (12)	
As	−0.41576 (4)	−0.38822 (4)	0.71249 (2)	0.01182 (10)	
O1	0.4878 (4)	−0.4176 (4)	0.68649 (4)	0.0247 (6)	
O2	−0.4350 (3)	−0.2496 (3)	0.73117 (4)	0.0154 (5)	
O3	−0.1870 (3)	−0.2856 (3)	0.70680 (5)	0.0217 (5)	
O4	0.4768 (3)	−0.1133 (3)	0.77606 (4)	0.0147 (5)	
H	−0.152 (5)	−0.354 (4)	0.7115 (6)	0.013 (10)*	

### Caesium indium bis[hydrogen arsenate(V)] (CsInHAsO42) Atomic displacement parameters (Å^2^)

**Table:**

	*U*^11^	*U*^22^	*U*^33^	*U*^12^	*U*^13^	*U*^23^
Cs1	0.0283 (2)	0.0283 (2)	0.0201 (3)	0.01415 (10)	0.000	0.000
Cs2	0.03461 (17)	0.03461 (17)	0.0190 (2)	0.01730 (8)	0.000	0.000
In1	0.01027 (13)	0.01027 (13)	0.00848 (17)	0.00513 (6)	0.000	0.000
In2	0.01173 (16)	0.01173 (16)	0.0075 (2)	0.00587 (8)	0.000	0.000
As	0.01466 (17)	0.01309 (17)	0.01022 (15)	0.00882 (14)	0.00082 (12)	0.00125 (11)
O1	0.0366 (15)	0.0361 (15)	0.0126 (11)	0.0265 (13)	−0.0067 (11)	−0.0014 (10)
O2	0.0155 (11)	0.0154 (11)	0.0157 (11)	0.0079 (9)	0.0056 (9)	−0.0020 (9)
O3	0.0174 (12)	0.0216 (13)	0.0290 (13)	0.0120 (11)	0.0083 (10)	0.0115 (11)
O4	0.0154 (11)	0.0126 (11)	0.0183 (11)	0.0086 (9)	−0.0032 (9)	−0.0057 (9)

### Caesium indium bis[hydrogen arsenate(V)] (CsInHAsO42) Geometric parameters (Å, º)

**Table:**

Cs1—O3	3.280 (3)	Cs2—O4^xii^	3.703 (2)
Cs1—O3^i^	3.280 (3)	Cs2—O4^xiii^	3.703 (2)
Cs1—O3^ii^	3.280 (3)	Cs2—O4^xiv^	3.703 (2)
Cs1—O3^iii^	3.280 (3)	Cs2—As^xi^	3.8762 (6)
Cs1—O3^iv^	3.280 (3)	Cs2—As^ix^	3.8762 (6)
Cs1—O3^v^	3.280 (3)	Cs2—As^x^	3.8762 (6)
Cs1—O2	3.434 (2)	In1—O2^xv^	2.127 (2)
Cs1—O2^ii^	3.434 (2)	In1—O2^iii^	2.127 (2)
Cs1—O2^v^	3.434 (2)	In1—O2^xvi^	2.127 (2)
Cs1—O2^iii^	3.434 (2)	In1—O4^xvii^	2.150 (2)
Cs1—O2^i^	3.434 (2)	In1—O4^iv^	2.150 (2)
Cs1—O2^iv^	3.434 (2)	In1—O4^xviii^	2.150 (2)
Cs1—H	3.44 (4)	In2—O1^vii^	2.133 (2)
Cs1—H^iv^	3.44 (4)	In2—O1^xi^	2.133 (3)
Cs1—H^iii^	3.44 (4)	In2—O1^xix^	2.133 (2)
Cs2—O3^iv^	3.121 (3)	In2—O1^iv^	2.133 (2)
Cs2—O3^iii^	3.121 (2)	In2—O1^xviii^	2.133 (3)
Cs2—O3	3.121 (3)	In2—O1^xvii^	2.133 (2)
Cs2—O1^vi^	3.419 (3)	As—O1^xx^	1.655 (2)
Cs2—O1^vii^	3.419 (3)	As—O2	1.671 (2)
Cs2—O1^viii^	3.419 (3)	As—O4^ii^	1.679 (2)
Cs2—O3^ix^	3.698 (3)	As—O3	1.743 (3)
Cs2—O3^x^	3.698 (3)	O3—H	0.83 (3)
Cs2—O3^xi^	3.698 (3)		
			
O3—Cs1—O3^i^	166.65 (9)	O1^vi^—Cs2—As^xi^	102.94 (4)
O3—Cs1—O3^ii^	119.30 (9)	O1^vii^—Cs2—As^xi^	88.69 (5)
O3^i^—Cs1—O3^ii^	69.82 (7)	O1^viii^—Cs2—As^xi^	25.24 (4)
O3—Cs1—O3^iii^	69.82 (7)	O3^ix^—Cs2—As^xi^	61.32 (4)
O3^i^—Cs1—O3^iii^	103.15 (9)	O3^x^—Cs2—As^xi^	94.72 (4)
O3^ii^—Cs1—O3^iii^	166.65 (10)	O3^xi^—Cs2—As^xi^	26.48 (4)
O3—Cs1—O3^iv^	69.82 (7)	O4^xii^—Cs2—As^xi^	70.49 (4)
O3^i^—Cs1—O3^iv^	119.30 (10)	O4^xiii^—Cs2—As^xi^	25.47 (3)
O3^ii^—Cs1—O3^iv^	103.15 (9)	O4^xiv^—Cs2—As^xi^	53.44 (4)
O3^iii^—Cs1—O3^iv^	69.82 (7)	O3^iv^—Cs2—As^ix^	107.26 (5)
O3—Cs1—O3^v^	103.15 (9)	O3^iii^—Cs2—As^ix^	174.10 (5)
O3^i^—Cs1—O3^v^	69.82 (7)	O3—Cs2—As^ix^	100.69 (5)
O3^ii^—Cs1—O3^v^	69.82 (7)	O1^vi^—Cs2—As^ix^	25.24 (4)
O3^iii^—Cs1—O3^v^	119.30 (9)	O1^vii^—Cs2—As^ix^	102.94 (4)
O3^iv^—Cs1—O3^v^	166.65 (9)	O1^viii^—Cs2—As^ix^	88.69 (5)
O3—Cs1—O2	47.28 (6)	O3^ix^—Cs2—As^ix^	26.48 (4)
O3^i^—Cs1—O2	119.81 (6)	O3^x^—Cs2—As^ix^	61.32 (4)
O3^ii^—Cs1—O2	126.64 (6)	O3^xi^—Cs2—As^ix^	94.72 (4)
O3^iii^—Cs1—O2	66.62 (6)	O4^xii^—Cs2—As^ix^	25.47 (3)
O3^iv^—Cs1—O2	111.83 (6)	O4^xiii^—Cs2—As^ix^	53.44 (4)
O3^v^—Cs1—O2	67.00 (6)	O4^xiv^—Cs2—As^ix^	70.49 (4)
O3—Cs1—O2^ii^	126.65 (6)	As^xi^—Cs2—As^ix^	78.314 (13)
O3^i^—Cs1—O2^ii^	66.61 (6)	O3^iv^—Cs2—As^x^	100.69 (5)
O3^ii^—Cs1—O2^ii^	47.28 (6)	O3^iii^—Cs2—As^x^	107.26 (5)
O3^iii^—Cs1—O2^ii^	119.81 (6)	O3—Cs2—As^x^	174.10 (5)
O3^iv^—Cs1—O2^ii^	67.01 (6)	O1^vi^—Cs2—As^x^	88.69 (5)
O3^v^—Cs1—O2^ii^	111.83 (6)	O1^vii^—Cs2—As^x^	25.24 (4)
O2—Cs1—O2^ii^	170.75 (8)	O1^viii^—Cs2—As^x^	102.94 (4)
O3—Cs1—O2^v^	67.01 (6)	O3^ix^—Cs2—As^x^	94.72 (4)
O3^i^—Cs1—O2^v^	111.83 (6)	O3^x^—Cs2—As^x^	26.48 (4)
O3^ii^—Cs1—O2^v^	66.61 (6)	O3^xi^—Cs2—As^x^	61.32 (4)
O3^iii^—Cs1—O2^v^	126.65 (6)	O4^xii^—Cs2—As^x^	53.44 (4)
O3^iv^—Cs1—O2^v^	119.81 (6)	O4^xiii^—Cs2—As^x^	70.49 (4)
O3^v^—Cs1—O2^v^	47.28 (6)	O4^xiv^—Cs2—As^x^	25.47 (4)
O2—Cs1—O2^v^	61.36 (8)	As^xi^—Cs2—As^x^	78.314 (13)
O2^ii^—Cs1—O2^v^	110.71 (3)	As^ix^—Cs2—As^x^	78.314 (13)
O3—Cs1—O2^iii^	111.83 (6)	O2^xv^—In1—O2^iii^	91.11 (9)
O3^i^—Cs1—O2^iii^	67.01 (6)	O2^xv^—In1—O2^xvi^	91.11 (9)
O3^ii^—Cs1—O2^iii^	119.81 (6)	O2^iii^—In1—O2^xvi^	91.11 (9)
O3^iii^—Cs1—O2^iii^	47.28 (6)	O2^xv^—In1—O4^xvii^	92.57 (9)
O3^iv^—Cs1—O2^iii^	66.61 (6)	O2^iii^—In1—O4^xvii^	175.38 (9)
O3^v^—Cs1—O2^iii^	126.65 (6)	O2^xvi^—In1—O4^xvii^	91.61 (9)
O2—Cs1—O2^iii^	110.71 (3)	O2^xv^—In1—O4^iv^	91.61 (8)
O2^ii^—Cs1—O2^iii^	77.59 (7)	O2^iii^—In1—O4^iv^	92.57 (9)
O2^v^—Cs1—O2^iii^	170.75 (8)	O2^xvi^—In1—O4^iv^	175.38 (9)
O3—Cs1—O2^i^	119.82 (6)	O4^xvii^—In1—O4^iv^	84.54 (9)
O3^i^—Cs1—O2^i^	47.28 (6)	O2^xv^—In1—O4^xviii^	175.38 (9)
O3^ii^—Cs1—O2^i^	111.83 (6)	O2^iii^—In1—O4^xviii^	91.61 (9)
O3^iii^—Cs1—O2^i^	67.01 (6)	O2^xvi^—In1—O4^xviii^	92.57 (9)
O3^iv^—Cs1—O2^i^	126.65 (6)	O4^xvii^—In1—O4^xviii^	84.54 (9)
O3^v^—Cs1—O2^i^	66.61 (6)	O4^iv^—In1—O4^xviii^	84.54 (9)
O2—Cs1—O2^i^	77.59 (7)	O2^xv^—In1—Cs2^xxi^	124.48 (6)
O2^ii^—Cs1—O2^i^	110.71 (3)	O2^iii^—In1—Cs2^xxi^	124.48 (6)
O2^v^—Cs1—O2^i^	110.71 (3)	O2^xvi^—In1—Cs2^xxi^	124.48 (6)
O2^iii^—Cs1—O2^i^	61.36 (8)	O4^xvii^—In1—Cs2^xxi^	50.96 (6)
O3—Cs1—O2^iv^	66.61 (6)	O4^iv^—In1—Cs2^xxi^	50.96 (6)
O3^i^—Cs1—O2^iv^	126.65 (6)	O4^xviii^—In1—Cs2^xxi^	50.96 (6)
O3^ii^—Cs1—O2^iv^	67.01 (6)	O1^vii^—In2—O1^xi^	94.56 (9)
O3^iii^—Cs1—O2^iv^	111.83 (6)	O1^vii^—In2—O1^xix^	94.55 (9)
O3^iv^—Cs1—O2^iv^	47.28 (6)	O1^xi^—In2—O1^xix^	94.55 (9)
O3^v^—Cs1—O2^iv^	119.81 (6)	O1^vii^—In2—O1^iv^	180.0
O2—Cs1—O2^iv^	110.71 (3)	O1^xi^—In2—O1^iv^	85.45 (9)
O2^ii^—Cs1—O2^iv^	61.36 (8)	O1^xix^—In2—O1^iv^	85.45 (9)
O2^v^—Cs1—O2^iv^	77.59 (7)	O1^vii^—In2—O1^xviii^	85.45 (9)
O2^iii^—Cs1—O2^iv^	110.71 (3)	O1^xi^—In2—O1^xviii^	180.0
O2^i^—Cs1—O2^iv^	170.75 (8)	O1^xix^—In2—O1^xviii^	85.45 (9)
O3—Cs1—H	13.9 (5)	O1^iv^—In2—O1^xviii^	94.55 (9)
O3^i^—Cs1—H	170.1 (7)	O1^vii^—In2—O1^xvii^	85.45 (9)
O3^ii^—Cs1—H	105.5 (5)	O1^xi^—In2—O1^xvii^	85.45 (9)
O3^iii^—Cs1—H	83.1 (5)	O1^xix^—In2—O1^xvii^	180.0
O3^iv^—Cs1—H	69.8 (6)	O1^iv^—In2—O1^xvii^	94.55 (9)
O3^v^—Cs1—H	100.5 (7)	O1^xviii^—In2—O1^xvii^	94.55 (9)
O2—Cs1—H	55.4 (6)	O1^vii^—In2—Cs2^xix^	56.94 (8)
O2^ii^—Cs1—H	117.1 (6)	O1^xi^—In2—Cs2^xix^	72.56 (8)
O2^v^—Cs1—H	58.5 (6)	O1^xix^—In2—Cs2^xix^	146.29 (7)
O2^iii^—Cs1—H	122.2 (6)	O1^iv^—In2—Cs2^xix^	123.06 (8)
O2^i^—Cs1—H	131.7 (6)	O1^xviii^—In2—Cs2^xix^	107.44 (8)
O2^iv^—Cs1—H	55.8 (6)	O1^xvii^—In2—Cs2^xix^	33.71 (7)
O3—Cs1—H^iv^	83.1 (5)	O1^vii^—In2—Cs2^xvii^	123.06 (8)
O3^i^—Cs1—H^iv^	105.5 (6)	O1^xi^—In2—Cs2^xvii^	107.44 (8)
O3^ii^—Cs1—H^iv^	100.5 (6)	O1^xix^—In2—Cs2^xvii^	33.71 (7)
O3^iii^—Cs1—H^iv^	69.8 (6)	O1^iv^—In2—Cs2^xvii^	56.94 (8)
O3^iv^—Cs1—H^iv^	13.9 (5)	O1^xviii^—In2—Cs2^xvii^	72.56 (8)
O3^v^—Cs1—H^iv^	170.2 (6)	O1^xvii^—In2—Cs2^xvii^	146.29 (7)
O2—Cs1—H^iv^	122.2 (6)	Cs2^xix^—In2—Cs2^xvii^	180.0
O2^ii^—Cs1—H^iv^	58.5 (6)	O1^vii^—In2—Cs2	33.71 (7)
O2^v^—Cs1—H^iv^	131.7 (6)	O1^xi^—In2—Cs2	123.06 (8)
O2^iii^—Cs1—H^iv^	55.8 (6)	O1^xix^—In2—Cs2	107.44 (8)
O2^i^—Cs1—H^iv^	117.1 (6)	O1^iv^—In2—Cs2	146.29 (7)
O2^iv^—Cs1—H^iv^	55.4 (6)	O1^xviii^—In2—Cs2	56.94 (8)
H—Cs1—H^iv^	83.7 (8)	O1^xvii^—In2—Cs2	72.56 (8)
O3—Cs1—H^iii^	69.8 (6)	Cs2^xix^—In2—Cs2	60.0
O3^i^—Cs1—H^iii^	100.5 (6)	Cs2^xvii^—In2—Cs2	120.0
O3^ii^—Cs1—H^iii^	170.2 (6)	O1^vii^—In2—Cs2^xvi^	107.44 (8)
O3^iii^—Cs1—H^iii^	13.9 (5)	O1^xi^—In2—Cs2^xvi^	33.71 (7)
O3^iv^—Cs1—H^iii^	83.1 (5)	O1^xix^—In2—Cs2^xvi^	123.06 (8)
O3^v^—Cs1—H^iii^	105.5 (5)	O1^iv^—In2—Cs2^xvi^	72.56 (8)
O2—Cs1—H^iii^	55.8 (6)	O1^xviii^—In2—Cs2^xvi^	146.29 (7)
O2^ii^—Cs1—H^iii^	131.7 (6)	O1^xvii^—In2—Cs2^xvi^	56.94 (8)
O2^v^—Cs1—H^iii^	117.1 (7)	Cs2^xix^—In2—Cs2^xvi^	60.0
O2^iii^—Cs1—H^iii^	55.4 (6)	Cs2^xvii^—In2—Cs2^xvi^	120.0
O2^i^—Cs1—H^iii^	58.5 (6)	Cs2—In2—Cs2^xvi^	120.0
O2^iv^—Cs1—H^iii^	122.2 (6)	O1^vii^—In2—Cs2^xxii^	72.56 (8)
H—Cs1—H^iii^	83.7 (8)	O1^xi^—In2—Cs2^xxii^	146.29 (7)
H^iv^—Cs1—H^iii^	83.7 (8)	O1^xix^—In2—Cs2^xxii^	56.94 (8)
O3^iv^—Cs2—O3^iii^	73.97 (8)	O1^iv^—In2—Cs2^xxii^	107.44 (8)
O3^iv^—Cs2—O3	73.97 (8)	O1^xviii^—In2—Cs2^xxii^	33.71 (7)
O3^iii^—Cs2—O3	73.97 (8)	O1^xvii^—In2—Cs2^xxii^	123.06 (8)
O3^iv^—Cs2—O1^vi^	82.81 (6)	Cs2^xix^—In2—Cs2^xxii^	120.0
O3^iii^—Cs2—O1^vi^	153.75 (6)	Cs2^xvii^—In2—Cs2^xxii^	60.0
O3—Cs2—O1^vi^	88.16 (7)	Cs2—In2—Cs2^xxii^	60.0
O3^iv^—Cs2—O1^vii^	88.16 (7)	Cs2^xvi^—In2—Cs2^xxii^	180.0
O3^iii^—Cs2—O1^vii^	82.80 (7)	O1^vii^—In2—Cs2^xxiii^	146.29 (7)
O3—Cs2—O1^vii^	153.75 (7)	O1^xi^—In2—Cs2^xxiii^	56.94 (8)
O1^vi^—Cs2—O1^vii^	108.90 (4)	O1^xix^—In2—Cs2^xxiii^	72.56 (8)
O3^iv^—Cs2—O1^viii^	153.75 (7)	O1^iv^—In2—Cs2^xxiii^	33.71 (7)
O3^iii^—Cs2—O1^viii^	88.16 (7)	O1^xviii^—In2—Cs2^xxiii^	123.06 (8)
O3—Cs2—O1^viii^	82.81 (6)	O1^xvii^—In2—Cs2^xxiii^	107.44 (8)
O1^vi^—Cs2—O1^viii^	108.90 (4)	Cs2^xix^—In2—Cs2^xxiii^	120.0
O1^vii^—Cs2—O1^viii^	108.90 (4)	Cs2^xvii^—In2—Cs2^xxiii^	60.0
O3^iv^—Cs2—O3^ix^	124.57 (9)	Cs2—In2—Cs2^xxiii^	180.0
O3^iii^—Cs2—O3^ix^	148.48 (9)	Cs2^xvi^—In2—Cs2^xxiii^	60.0
O3—Cs2—O3^ix^	86.50 (7)	Cs2^xxii^—In2—Cs2^xxiii^	120.0
O1^vi^—Cs2—O3^ix^	44.45 (6)	O1^xx^—As—O2	117.25 (12)
O1^vii^—Cs2—O3^ix^	119.69 (6)	O1^xx^—As—O4^ii^	108.41 (12)
O1^viii^—Cs2—O3^ix^	64.61 (6)	O2—As—O4^ii^	113.77 (11)
O3^iv^—Cs2—O3^x^	86.50 (7)	O1^xx^—As—O3	105.44 (13)
O3^iii^—Cs2—O3^x^	124.56 (9)	O2—As—O3	104.33 (12)
O3—Cs2—O3^x^	148.48 (8)	O4^ii^—As—O3	106.72 (11)
O1^vi^—Cs2—O3^x^	64.61 (6)	O1^xx^—As—Cs2^ix^	61.74 (9)
O1^vii^—Cs2—O3^x^	44.45 (6)	O2—As—Cs2^ix^	174.14 (8)
O1^viii^—Cs2—O3^x^	119.69 (6)	O4^ii^—As—Cs2^ix^	71.50 (8)
O3^ix^—Cs2—O3^x^	84.57 (6)	O3—As—Cs2^ix^	71.06 (9)
O3^iv^—Cs2—O3^xi^	148.48 (9)	O1^xx^—As—Cs1	139.73 (11)
O3^iii^—Cs2—O3^xi^	86.50 (7)	O2—As—Cs1	55.91 (8)
O3—Cs2—O3^xi^	124.57 (9)	O4^ii^—As—Cs1	109.82 (8)
O1^vi^—Cs2—O3^xi^	119.69 (6)	O3—As—Cs1	51.15 (9)
O1^vii^—Cs2—O3^xi^	64.61 (6)	Cs2^ix^—As—Cs1	120.598 (10)
O1^viii^—Cs2—O3^xi^	44.45 (6)	O1^xx^—As—Cs2	75.26 (11)
O3^ix^—Cs2—O3^xi^	84.57 (6)	O2—As—Cs2	99.50 (8)
O3^x^—Cs2—O3^xi^	84.57 (6)	O4^ii^—As—Cs2	138.02 (8)
O3^iv^—Cs2—O4^xii^	113.69 (6)	O3—As—Cs2	37.36 (8)
O3^iii^—Cs2—O4^xii^	159.43 (6)	Cs2^ix^—As—Cs2	74.638 (9)
O3—Cs2—O4^xii^	126.02 (6)	Cs1—As—Cs2	68.078 (14)
O1^vi^—Cs2—O4^xii^	44.41 (5)	As^xxiv^—O1—In2^xxv^	140.67 (15)
O1^vii^—Cs2—O4^xii^	78.54 (5)	As^xxiv^—O1—Cs2^vi^	93.02 (10)
O1^viii^—Cs2—O4^xii^	89.74 (5)	In2^xxv^—O1—Cs2^vi^	126.03 (9)
O3^ix^—Cs2—O4^xii^	43.56 (5)	As^xxiv^—O1—Cs2^xxiv^	82.42 (10)
O3^x^—Cs2—O4^xii^	41.47 (5)	In2^xxv^—O1—Cs2^xxiv^	97.96 (9)
O3^xi^—Cs2—O4^xii^	77.76 (5)	Cs2^vi^—O1—Cs2^xxiv^	80.74 (5)
O3^iv^—Cs2—O4^xiii^	159.43 (6)	As^xxiv^—O1—Cs2^xxv^	118.31 (12)
O3^iii^—Cs2—O4^xiii^	126.02 (6)	In2^xxv^—O1—Cs2^xxv^	82.33 (8)
O3—Cs2—O4^xiii^	113.70 (7)	Cs2^vi^—O1—Cs2^xxv^	72.50 (5)
O1^vi^—Cs2—O4^xiii^	78.54 (5)	Cs2^xxiv^—O1—Cs2^xxv^	146.39 (6)
O1^vii^—Cs2—O4^xiii^	89.74 (6)	As—O2—In1^xxvi^	122.05 (12)
O1^viii^—Cs2—O4^xiii^	44.41 (5)	As—O2—Cs1	100.33 (9)
O3^ix^—Cs2—O4^xiii^	41.47 (5)	In1^xxvi^—O2—Cs1	125.68 (9)
O3^x^—Cs2—O4^xiii^	77.76 (5)	As—O2—Cs2	60.78 (7)
O3^xi^—Cs2—O4^xiii^	43.56 (5)	In1^xxvi^—O2—Cs2	162.87 (9)
O4^xii^—Cs2—O4^xiii^	45.97 (6)	Cs1—O2—Cs2	66.29 (4)
O3^iv^—Cs2—O4^xiv^	126.02 (6)	As—O3—Cs2	122.83 (12)
O3^iii^—Cs2—O4^xiv^	113.69 (6)	As—O3—Cs1	104.40 (11)
O3—Cs2—O4^xiv^	159.43 (6)	Cs2—O3—Cs1	94.64 (7)
O1^vi^—Cs2—O4^xiv^	89.74 (5)	As—O3—Cs2^ix^	82.46 (10)
O1^vii^—Cs2—O4^xiv^	44.41 (5)	Cs2—O3—Cs2^ix^	93.50 (7)
O1^viii^—Cs2—O4^xiv^	78.53 (5)	Cs1—O3—Cs2^ix^	164.05 (8)
O3^ix^—Cs2—O4^xiv^	77.76 (5)	As—O3—H	107 (3)
O3^x^—Cs2—O4^xiv^	43.56 (5)	Cs2—O3—H	124 (3)
O3^xi^—Cs2—O4^xiv^	41.47 (5)	Cs1—O3—H	94 (3)
O4^xii^—Cs2—O4^xiv^	45.97 (6)	Cs2^ix^—O3—H	70 (3)
O4^xiii^—Cs2—O4^xiv^	45.97 (6)	As^ii^—O4—In1^xxv^	129.32 (12)
O3^iv^—Cs2—As^xi^	174.10 (5)	As^ii^—O4—Cs2^xxvii^	83.03 (8)
O3^iii^—Cs2—As^xi^	100.69 (5)	In1^xxv^—O4—Cs2^xxvii^	102.24 (8)
O3—Cs2—As^xi^	107.26 (5)		

Symmetry codes: (i) *x*−*y*, −*y*, −*z*+3/2; (ii) −*x*, −*x*+*y*, −*z*+3/2; (iii) −*x*+*y*, −*x*, *z*; (iv) −*y*, *x*−*y*, *z*; (v) *y*, *x*, −*z*+3/2; (vi) −*x*+2/3, −*y*−2/3, −*z*+4/3; (vii) *y*+2/3, −*x*+*y*+4/3, −*z*+4/3; (viii) *x*−*y*−4/3, *x*−2/3, −*z*+4/3; (ix) −*x*−1/3, −*y*−2/3, −*z*+4/3; (x) *y*+2/3, −*x*+*y*+1/3, −*z*+4/3; (xi) *x*−*y*−1/3, *x*+1/3, −*z*+4/3; (xii) *x*−1/3, *x*−*y*−2/3, *z*−1/6; (xiii) −*y*−1/3, −*x*+1/3, *z*−1/6; (xiv) −*x*+*y*+2/3, *y*+1/3, *z*−1/6; (xv) −*y*, *x*−*y*+1, *z*; (xvi) *x*+1, *y*+1, *z*; (xvii) *x*, *y*+1, *z*; (xviii) −*x*+*y*+1, −*x*+1, *z*; (xix) −*x*+2/3, −*y*+1/3, −*z*+4/3; (xx) *x*−1, *y*, *z*; (xxi) −*y*+1/3, −*x*+2/3, *z*+1/6; (xxii) −*x*−1/3, −*y*+1/3, −*z*+4/3; (xxiii) −*x*+2/3, −*y*+4/3, −*z*+4/3; (xxiv) *x*+1, *y*, *z*; (xxv) *x*, *y*−1, *z*; (xxvi) *x*−1, *y*−1, *z*; (xxvii) −*y*+1/3, −*x*−1/3, *z*+1/6.

### Caesium indium bis[hydrogen arsenate(V)] (CsInHAsO42) Hydrogen-bond geometry (Å, º)

**Table:**

*D*—H···*A*	*D*—H	H···*A*	*D*···*A*	*D*—H···*A*
O3—H···O4^xxviii^	0.83 (3)	1.80 (3)	2.621 (3)	170 (4)

Symmetry code: (xxviii) *y*, *x*−1, −*z*+3/2.
